# Rrp12 and the Exportin Crm1 Participate in Late Assembly Events in the Nucleolus during 40S Ribosomal Subunit Biogenesis

**DOI:** 10.1371/journal.pgen.1004836

**Published:** 2014-12-04

**Authors:** Giulia Moriggi, Blanca Nieto, Mercedes Dosil

**Affiliations:** 1 Centro de Investigación del Cáncer and Instituto de Biología Molecular y Celular del Cáncer (IBMCC), CSIC-University of Salamanca, Salamanca, Spain; 2 Departamento de Bioquímica y Biología Molecular, University of Salamanca, Salamanca, Spain; University of Regensburg Germany, Germany

## Abstract

During the biogenesis of small ribosomal subunits in eukaryotes, the pre-40S particles formed in the nucleolus are rapidly transported to the cytoplasm. The mechanisms underlying the nuclear export of these particles and its coordination with other biogenesis steps are mostly unknown. Here we show that yeast Rrp12 is required for the exit of pre-40S particles to the cytoplasm and for proper maturation dynamics of upstream 90S pre-ribosomes. Due to this, in vivo elimination of Rrp12 leads to an accumulation of nucleoplasmic 90S to pre-40S transitional particles, abnormal 35S pre-rRNA processing, delayed elimination of processing byproducts, and no export of intermediate pre-40S complexes. The exportin Crm1 is also required for the same pre-ribosome maturation events that involve Rrp12. Thus, in addition to their implication in nuclear export, Rrp12 and Crm1 participate in earlier biosynthetic steps that take place in the nucleolus. Our results indicate that, in the 40S subunit synthesis pathway, the completion of early pre-40S particle assembly, the initiation of byproduct degradation and the priming for nuclear export occur in an integrated manner in late 90S pre-ribosomes.

## Author Summary

During the synthesis of small ribosomal subunits in eukaryotes, the pre-40S particles formed in the nucleolus are rapidly transported to the cytoplasm. The mechanisms involved in the nuclear export of these particles and its coordination with other steps of the 40S synthesis pathway are mostly unknown. In this work we studied the function of Rrp12, the only major non-ribosomal factor of nuclear pre-40S particles that does not remain stably associated to them during maturation in the cytoplasm. We demonstrate that Rrp12 is required for the exit of pre-40S particles to the cytoplasm. Remarkably, we also found that Rrp12, together with the Crm1 exportin, participates in processes that occur in early pre-ribosomes in the nucleolus, including the processing of the pre-rRNA and the elimination of processing byproducts. Thus, Rrp12 and Crm1 participate in maturation steps that take place upstream of nuclear export. Our results indicate that, in the 40S subunit synthesis pathway, the completion of early pre-40S particle assembly, the initiation of byproduct degradation and the priming for nuclear export occur in an integrated manner in nucleolar pre-ribosomes.

## Introduction

**Figure 1:**
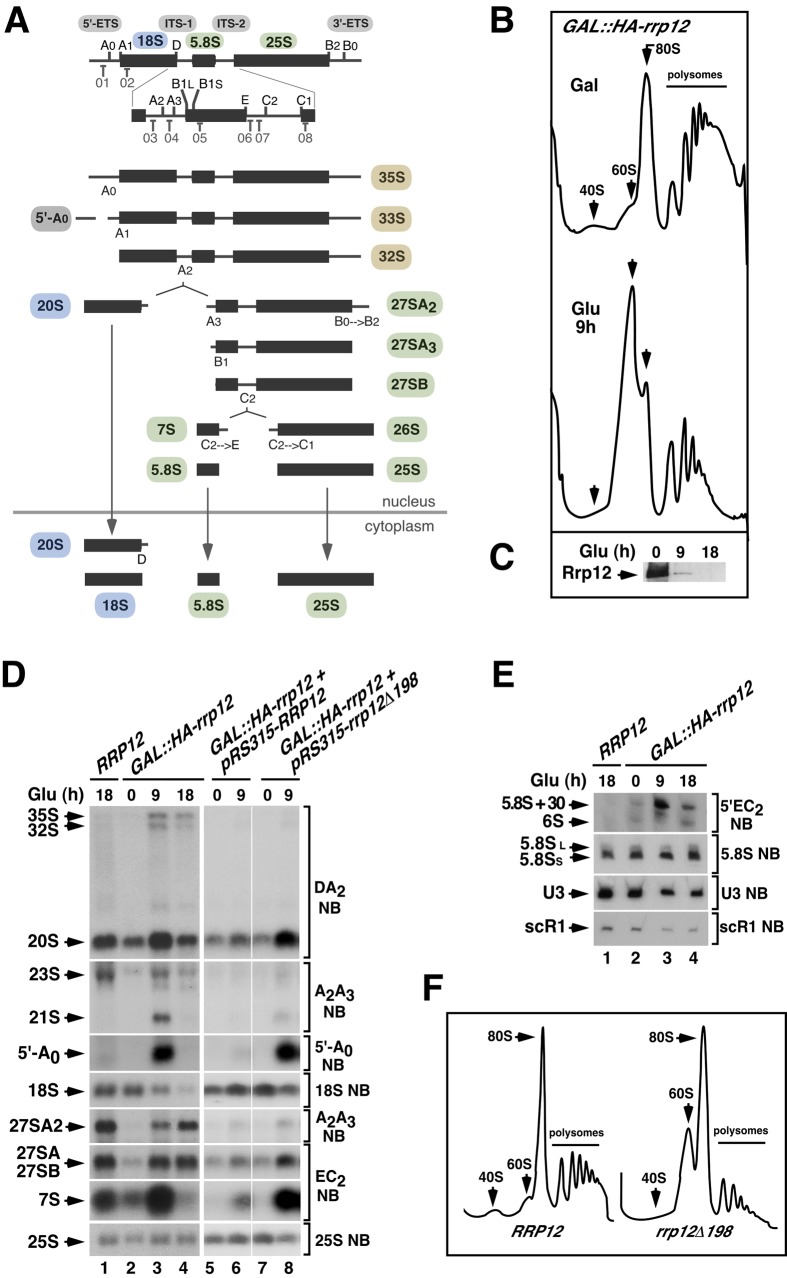
Defects in Rrp12 function block the synthesis of 40S subunits but not of 60S subunits. (**A**) Structure of the 35S pre-rRNA and major intermediates of the rRNA processing pathway. The names of the initial pre-rRNA species are highlighted in brown. Those for the 18S pre-rRNA precursor, 5.8S/25S precursors, and 5′-A_0_ processing byproduct are highlighted in blue, green and grey, respectively. For simplicity, an alternative pathway to form 27SB_L_ pre-rRNA is not shown. Binding sites for oligonucleotide probes (01 to 08) used in Northern blot experiments are indicated in the upper diagram. Those included probe 03 for the DA_2_ region, probe 04 for the A_2_–A_3_ region, probe 01 for the 5′-A_0_ region, probe 02 for the 18S region, probe 07 for the E-C_2_ region, probe 08 for the 25S region, probe 06 for the 5′EC_2_ region and probe 05 for the 5.8S region. (**B**) Sucrose-gradient sedimentation analysis of ribosomal fractions (40S, 60S, 80S and polysomes) of cell lysates from *GAL::HA-rrp12* cells that have been grown in galactose (Gal)-containing media or shifted to a glucose (Glu)-containing media for 9 hours. Depletion of Rrp12 protein was analyzed by Western blot (**C**). (**D** and **E**) Northern blot (NB) analysis of total RNAs extracted from *RRP12*, *GAL::HA-rrp12*, and *GAL::HA-rrp12* cells containing plasmids encoding either wild type Rrp12 or the hypomorphic Rrp12 (deletion Δ1-198) mutant. Cells were grown at 30°C in galactose-containing media and shifted to glucose-containing media for the indicated times. The specific region of the 35S pre-rRNA recognized by the Northern blot probe is indicated on the right. This will be similarly indicated in the rest of analyses presented in this work. The thin white lines between lanes 6 and 7 indicate the presence of in-between lanes in the same blot that have been removed. The experiment shown in E also includes, as a loading control, the RNA of the signal recognition particle scR1. (**F**) Sucrose-gradient sedimentation analysis of ribosomal fractions (40S, 60S, 80S and polysomes) of cell lysates from *GAL::HA-rrp12* cells containing plasmids encoding either wild type Rrp12 or the hypomorphic Rrp12 (Δ1-198) mutant. Cells were grown continuously in glucose-containing media.

The formation of ribosomes in eukaryotic cells requires the production and subsequent assembly of four rRNAs and ≈80 ribosomal proteins into small (40S) and large (60S) ribosome subunits. In the yeast *S. cerevisiae*, three out of those four rRNAs (18S, 5.8S and 25S) are transcribed together in the nucleolus in the context of a common polycistronic 35S pre-rRNA (see scheme in **Fig. 1A**) [1], [2]. This primary rRNA precursor is bound by ribosomal proteins, as well as by the U3 small nucleolar ribonucleoprotein (snoRNP) and ≈70 non-ribosomal factors, to form the large 90S pre-ribosomal particle [3]. This process involves the recruitment of smaller multiprotein subunits that associate to the nascent transcript in a stepwise manner [4], [5], [6]. The 35S pre-RNA then undergoes serial cleavages at the A_0_, A_1_ and A_2_ sites to generate the 20S and 27SA_2_ pre-rRNAs (**Fig. 1A**) [1], [2]. These three cleavages can also occur co-transcriptionally within the so-called small subunit (SSU) processome, a complex very similar in composition to the 90S pre-ribosome [7], [8]. The disassembly of the 90S pre-ribosome leads to the formation of pre-40S and pre-60S particles containing the 20S and the 27SA_2_ pre-rRNAs, respectively [2], [9]. This process is accompanied by the release of the non-ribosomal components originally present in the 90S pre-ribosome and the rapid degradation of processing byproducts [2].

The early pre-60S particles contain >40 associated factors, and undergo multiple maturation steps that are accompanied by major changes in composition until exiting the nucleus [2], [10], [11], [12]. In contrast, the early pre-40S particles are thought to have a relatively low compositional complexity and are rapidly exported, consistent with the fact that the 20S pre-rRNA is not further processed inside the nucleus (**Figure 1A**) [2], [10], [11], [13]. Final maturation of 40S subunits, which includes a proofreading step through association to 60S subunits and the cleavage of the 20S pre-rRNA at site D, takes place in the cytoplasm [14], [15], [16], [17]. Due to the rapid kinetics of transit thorough the nucleoplasm, it is assumed that the major events of pre-40S particle assembly take place concurrently with the 35S pre-RNA cleavage steps in the nucleolus. Despite this, the pre-40S particles released from 90S pre-ribosomes have to undergo some transformations before leaving the nucleus. These include the recruitment of factors that will participate in cytoplasmic maturation processes as well as transport proteins involved in particle transit through nuclear pores [18], [19]. Pre-40S particles are also known to undergo a kinase-dependent conformational rearrangement that might be required for nuclear export [20].

Despite the significant progress in the understanding of the compositional changes that take place between 90S and pre-40S pre-ribosomes, there are still many questions about the nucleolar assembly and nuclear maturation of 40S subunits that remain unanswered. For example, it is still unclear how the early pre-40S particles are assembled within the 90S pre-ribosome and how similar they are, at the structural level, to the pre-40S particles that reach the cytoplasm. It is also unknown how and when pre-40S particles become competent for export, and how the export process itself takes place. The Ran GTPase and the Crm1 exportin are both essential for pre-40S particles to exit the nucleus [19], [21], but the factors or mechanisms that mediate their interaction with those particles are not known. Tackling these questions has been difficult so far due to the large number of components involved, the transient nature of nucleoplasmic transit and nuclear exit, and the lack of success in dissecting these activities in separable or mechanistically simple steps.

Rrp12 is a karyopherin-like protein previously described as essential for the export of pre-40S and pre-60S particles out of the nucleus [22]. In addition, Rrp12 has been found to facilitate ribosome-unrelated nuclear import processes [23]. In relation with the role of Rrp12 in pre-ribosome export, it is presently unclear whether such function is due to an implication in the assembly of pre-ribosomal complexes, their maturation in the nucleus, the actual transport event, or compound roles in some of the above processes. To address those issues, we studied in detail the consequences of a partial or total loss of function of Rrp12 in *S. cerevisiae*. Our results show that Rrp12 is required for nuclear export of pre-40S particles. However, and unlike previously-published observations, we found that Rrp12 is not essential for 60S subunit production. During the course of our experiments, we additionally uncovered that this protein, together with the Crm1 exportin, is important for the last processing events of the 35S pre-rRNA within the 90S pre-ribosome, and for the rapid elimination of the 5′-A_0_ byproduct. The characterization of these new roles indicates that the completion of assembly and the nuclear export of the pre-40S particle are intertwined processes.

## Results

### Rrp12 is primarily required for the synthesis of 40S ribosomal subunits

A previous report described that Rrp12 was required for export of both pre-40S and 60S ribosomal subunits from the nucleus to the cytoplasm [22]. However, we observed using a yeast strain with the *RRP12* gene under a galactose-inducible promoter (*GAL::HA-rrp12*) that this protein was specifically involved in the biosynthesis of 40S subunits. Evidence in favor of such conclusion included: **(i)** Polysome profile analyses showing that the loss of Rrp12 was associated with reductions in the content of free 40S subunits and polysomes, but not of free 60S subunits (**Figure 1B** and **Figure 1C**). In fact, the relative abundance of the large subunits was clearly increased in the absence of Rrp12 (**Figure 1B** and **Figure 1C**). **(ii)** Northern blot analyses demonstrating a decrease in the steady-state amount of the 18S rRNA (present in 40S subunits) but not in those of the 5.8S and 25S rRNAs (present in 60S subunits) in Rrp12-depleted cells (**Figure 1D**, left panels; and **Figure 1E**). Such alterations were found to be associated with an increase in the abundance of the 20S pre-rRNA, the immediate upstream precursor for the 18S rRNA (**Figure 1D**; see scheme in **Figure 1A**), indicating that the cleavage at site D is inhibited. Consistent with previously published data [22], we also observed some accumulation of the 35S and 32S pre-rRNAs, a reduction in the content of the 27SA_2_ pre-rRNA, and the generation of the aberrant 21S pre-rRNA (a species produced from direct cleavage of the 32S pre-rRNA at site A_3_) (**Figure 1D**; see scheme in **Figure 1A**). These results indicate that, in addition to the major defect in the cleavage at site D, the loss of Rrp12 causes partial defects in the early cleavages at sites A_0_ and A_1_ and, to a larger extent, at site A_2_. We also detected a delay in the processing events of 5.8S rRNA precursors manifested by the presence of both the 7S pre-rRNA and aberrant 3′-extended forms of the 5.8S rRNA (5.8S+30) in Rrp12-depleted cells (**Figure 1D** and **Figure 1E**). Curiously, we found that the absence of Rrp12 led to an increase in the abundance of the 5′-A_0_ fragment (**Figure 1D**), a byproduct produced when the rRNA precursor is cleaved at site A_0_ (**Figure 1A**). Similar defects, although milder in intensity, were observed in a constitutive manner when pre-rRNA analyses were performed in a yeast strain (*rrp12-Δ198*) expressing a hypomorphic version of Rrp12 (**Figure 1D**, right panels; **Figure 1F**). Taken together, these data indicate that Rrp12 is absolutely required for the generation of the 18S rRNA from 20S pre-rRNA and, in addition, important for both the rapid elimination of the 5′-A_0_ fragment and the normal processing of both 32S and 5.8S pre-rRNA precursors. Despite this latter function, Rrp12 does not seem to have any major influence on the overall production of 60S ribosomal subunits.

### Rrp12 is present in both 90S and pre-40S particles

**Figure 2:**
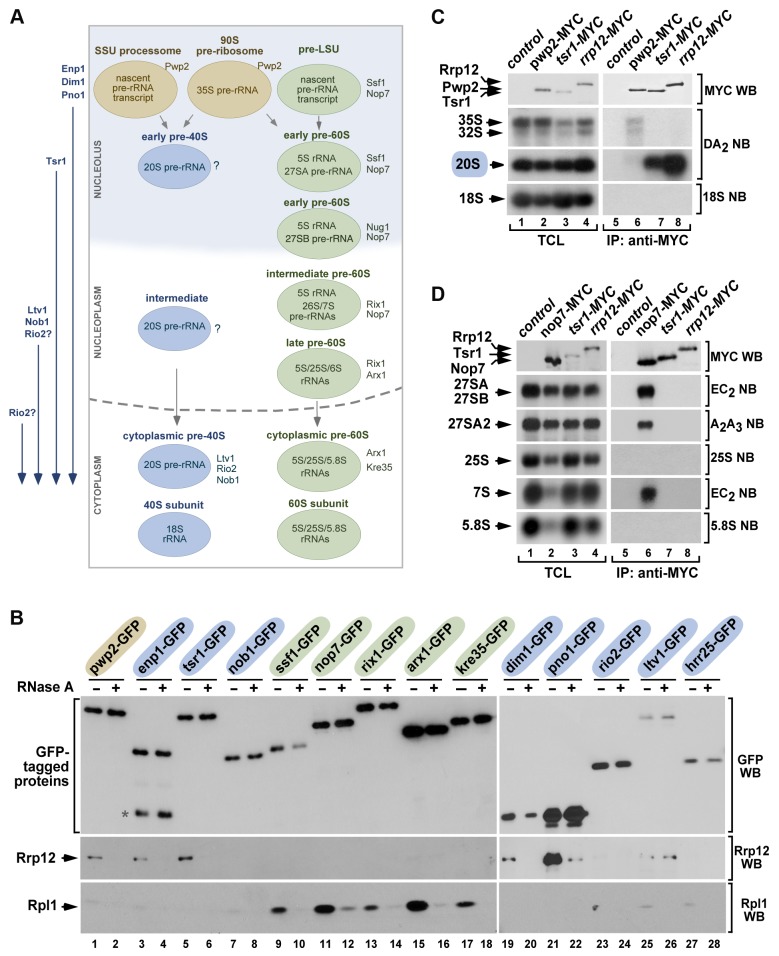
Rrp12 is present in both 90S pre-ribosomes and pre-40S particles. (**A**) Scheme of the maturation of pre-ribosomes. The names of specific factors frequently used for purifying each pre-ribosome are indicated on the right. In rapidly growing cells, ∼60% of primary transcripts are cleaved at A_0_–A_1_–A_2_ co-transcriptionally within the small subunit (SSU) processome and, after this, the precursor of the large subunit (pre-LSU) is assembled onto the nascent pre-rRNA. When not cleaved co-transcriptionally, the full-length 35S pre-rRNA is assembled into the 90S pre-ribosome, a particle very similar to the SSU-processome. The order of incorporation of the seven major maturation factors present in cytoplasmic pre-40S particles is shown on the left. Enp1, Dim1 and Pno1 are recruited to 90S/SSU particles. Tsr1 is recruited to early pre-40S particles in the nucleolus. Ltv1 and Nob1 join pre-40S particles in the nucleus. The step of incorporation of Rio2 remains ill defined. (**B**) Western blot analysis showing coimmunoprecipitation of Rrp12 (second panels from top) and of the control protein Rpl1 (bottom panels) with the indicated 90S pre-ribosome and nuclear pre-40S factors (top) in the presence (+) or absence (−) of RNase A in cell lysates. Factors present in 90S, pre-40S and pre-60S particles are shaded in brown, blue and green, respectively. The amount of GFP-Trap purified bait is shown in the first panels from top. The asterisk indicates a protein species in the Enp1-GFP purification lane that probably corresponds to a partial degradation product. (**C** and **D**) Northern blot analysis showing coimmunoprecipitation of pre-rRNA species (second to bottom panels on the right) with the indicated MYC-tagged proteins in normal cells. As control, parallel Northern blots were performed on total RNAs prepared from the same total cell lysate samples used for the immunoprecipitations (second to bottom panels on the left). Western blot experiments were performed to analyze the amount of MYC-tagged protein present in the total cell lysates (top panel on the left) and immunoprecipitations (top panel on the right). TCL, total cell lysates. IP, immunoprecipitation.

Our group and others have previously shown that Rrp12 copurifies with components of 90S and pre-40S particles [3], [18], [22], [23]. However, there is no detailed information about its relative content in different subsets of pre-40S complexes. Using coimmunoprecipitation experiments, we observed that endogenous Rrp12 interacted with green fluorescent protein (GFP)-tagged versions of factors present in nucleolar 90S (Pwp2, Enp1, Dim1, Pno1; **Figure 2A** and **Figure 2B**, lanes 1 to 4 and lanes 19 to 22) and nucleoplasmic pre-40S (Enp1, Dim1, Pno1, Tsr1; **Figure 2A** and **Figure 2B**, lanes 3 to 6 and lanes 19 to 22) particles. These interactions took place within the context of ribonucleproteic complexes, because they were eliminated by RNase treatment (**Figure 2B**, lanes 1 to 6 and lanes 19 to 22). By contrast, we did not detect any association of Rrp12 in these experiments with either Nob1 or Rio2, two proteins mostly present in cytoplasmic pre-40S particles (**Figure 2A** and **Figure 2B**, lanes 7,8 and lanes 23,24). Rrp12 did show an interaction with Ltv1, a protein that, like Nob1 and Rio2, is mainly detected in cytoplasmic pre-40S complexes (**Figure 2A** and **Figure 2B**, lanes 25,26). This interaction is the only one that cannot be disrupted by RNase treatment (**Figure 2B**, lanes 25,26), indicating that it survives pre-40S particle disassembly or, alternatively, that takes place outside those particles. In agreement with the results presented in **Figure 1**, we could not detect interactions of Rrp12 with proteins present in early (Ssf1, Nop7; **Figure 2A** and **Figure 2B**, lanes 9 to 12), intermediate nuclear (Rix1; **Figure 2A** and **Figure 2B**, lanes 13,14), late nuclear (Arx1; **Figure 2A** and **Figure 2B**, lanes 15,16) or cytoplasmic (Kre35; **Figure 2A** and **Figure 2B**, lanes 17,18) pre-60S complexes. These results suggest that Rrp12 is predominantly associated to both nucleolar and nuclear pre-40S pre-ribosomes while it is weakly associated, or not bound at all, to the cytoplasmic ones. Further analyses of Rrp12-MYC immunoprecipitates by Northern blot confirmed the predominant presence of this protein in the 40S synthesis pathway and, in addition, evidenced that its interactions with nucleolar and nucleoplasmic particles exhibit differential features. Thus, we observed that the association of Rrp12 to pre-40S particles had to be rather strong, as inferred by the stable coimmunoprecipiation of the 20S pre-rRNA with Rrp12-MYC (**Figure 2C**). Indeed, the amount of this pre-RNA in those complexes is even higher than that seen in the case of immunoprecipitations performed with Tsr1, a factor that stably associates with both nucleolar- and cytoplasmic-located pre-40S particles (**Figure 2A** and **Figure 2C**). By contrast, we could not detect any significant amount of 35S and 32S pre-RNAs in the Rrp12-MYC immunoprecipitates, suggesting that the association with the 90S particle is either labile or restricted to a minor pool of Rrp12-containing complexes (**Figure 2C**). As control, we found that these two pre-RNAs do coimmunoprecipitate with Pwp2 (**Figure 2C**), an integral component of the 90S pre-ribosome (**Figure 2A**). Consistent with the lack of Rrp12 in the purifications of pre-60S complexes (see above **Figure 2B**), we could not observe any interaction of Rrp12-MYC with the 27S or 7S pre-rRNAs. As expected, these two pre-rRNAs do coimmunoprecipitate with the early pre-60S particle component Nop7-MYC (**Figure 2D**, see scheme in **Figure 2A**). These results indicate that Rrp12 does not stably associate with pre-60S particles.

### Rrp12 is not required for pre-40S particle assembly

**Figure 3:**
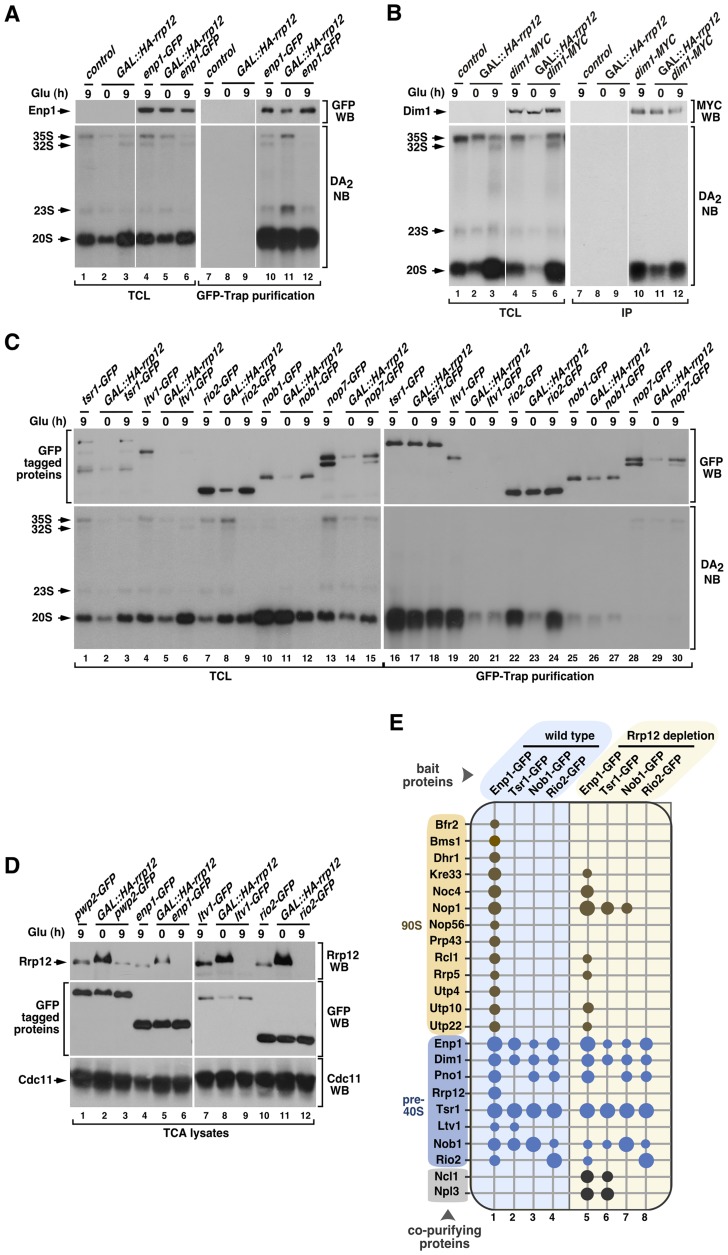
Rrp12 is not involved in pre-40S particle assembly. (**A** to **C**) Bottom panels, Northern blot analysis showing coimmunoprecipitation of the 20S pre-rRNA with Enp1-GFP (A), Dim1-MYC (B), Tsr1-GFP (C), Ltv1-GFP (C), Rio2-GFP (C), Nob1-GFP (C) and Nop7-GFP (C) before (0) and upon depletion of Rrp12 for 9 hours. Top panels, Western blot analysis showing the amount of immunoprecipitated proteins in these experiments. Mobility of pre-RNA species is indicated on the left of each bottom panel. Antibodies used in the immunoblots and Northern blot probes are shown on the right of the top and bottom panels, respectively. The thin white lines between lanes 3 and 4, and 9 and 10, shown in A and B, indicate the presence of in-between lanes in the same blot that have been removed. (**D**) Western blot analyses of trichloroacetic acid (TCA) precipitated cell lysates showing the amount of Rrp12 (top panels) and the indicated GFP-tagged proteins (middle panels) under the indicated growth conditions. The amount of Cdc11 was used as loading control (bottom panel). (**E**) Pre-ribosomal factors (listed on the left) copurifying with the indicated GFP-tagged proteins (top) in the presence (columns 1 to 4) or absence (columns 5 to 8) of Rrp12. Copurification of a factor with the bait is indicated with a dot. For Rrp12 depletion, *GAL::HA-rrp12* cells were shifted from galactose-containing media to glucose-containing media for 12 hours. The pre-ribosomal particles that contain the prey proteins are indicated on the left. Size of dots represents the relative amount of coimmunoprecipitated protein in each case (see Materials and Methods).

We next focused on the cause of the block in the maturation of 20S pre-rRNA to 18S rRNA found in Rrp12-depleted cells. Given the restricted presence of Rrp12 to nucleolar 90S and nucleoplasmic pre-40S complexes, this phenotype could be due to defects in the assembly of the pre-40S particle inside the nucleus. However, this does not seem to be the case because the depletion of Rrp12 does not affect the stability of both early and late nuclear pre-40S components (Enp1, Dim1, Tsr1, Rio2, Nob1; **Figures 3A** to **3C**; top panels). Likewise, it does not block the interaction of those proteins with the 20S pre-rRNA (**Figures 3A** to **3C**; bottom panels). However, the depletion of Rrp12, although not affecting the steady state levels of Ltv1 in cell lysates prepared by TCA precipitation (**Figure 3D**, compare lanes 7 and 9), does cause a destabilization of that protein under the conditions used for the pre-rRNA coimmunoprecipitation analyses (**Figure 3C**; top panel, compare lanes 4 and 6). Such behavior may reflect a functional relationship of Rrp12 and Ltv1 in vivo, because we observed using sucrose gradient fractionation experiments that the loss of Rrp12 leads to a substantial decrease in the amount of Ltv1 that is stably incorporated onto ∼40S complexes (**Figure S1A**). This effect is specific, because the depletion of Rrp12 does not affect the incorporation of both Enp1 and Rio2 onto those complexes (**Figure S1B** and **Figure S1C**).

Mass spectrometry experiments further confirmed that the absence of Rrp12 does not have a major effect in the composition of pre-40S complexes. Indeed, we found that both the pattern and strength of the associations exhibited by four pre-40S factors (Enp1, Tsr1, Nob1 and Rio2) with the rest of major pre-40S particle components are quite similar to those observed in wild-type cells (**Figure 3E**, compare columns 1 to 4 with columns 5 to 8). The only exception observed is the loss of the interaction of both Enp1 and Tsr1 with Ltv1 (**Figure 3E**, compare columns 1 and 2 with columns 5 and 6), a defect probably derived from the impaired recruitment of Ltv1 to the pre-40S particle seen in above experiments. Interestingly, we observed that the loss of Rrp12 promotes the formation of new interactions of both Enp1 and Tsr1 with the tRNA methyltransferase Ncl1 and the abundant hnRNP protein Npl3 (**Figure 3E**, compare columns 1 and 2 with columns 5 and 6). Likewise, Tsr1 and Nob1 interact with the 90S particle component Nop1 (**Figure 3E**, compare columns 2 and 3 with columns 6 and 7). These results indicate that Rrp12 is not required for the formation of the core structure of the pre-40S particle, although it may contribute to the release of specific nucleolar factors such as Nop1. In addition, they show that Rrp12 appears to be dispensable for the recruitment of some factors with hitherto unknown roles in the synthesis of 40S subunits (i.e., Ncl1, Npl3). Also consistent with a correct particle assembly in the absence of Rrp12, we found using Western blot analyses that Prp43 and Mex67 [24], [25], [26], [27], two factors that are not usually detected in this type of proteomics analysis due to their weak interaction with pre-40S particles, remain particle-associated in the absence of Rrp12 (**Figure S2**). Interestingly, the absence of Rrp12 does promote a reduction in the association of Enp1 with some, but not all, of its usual partners within the 90S particle (**Figure 3E**, compare columns 1 and 5). These data indicate that the lack of Rrp12 may affect either the composition or maturation dynamics of 90S pre-ribosomes.

### Rrp12 is required for nuclear export of pre-40S particles

**Figure 4:**
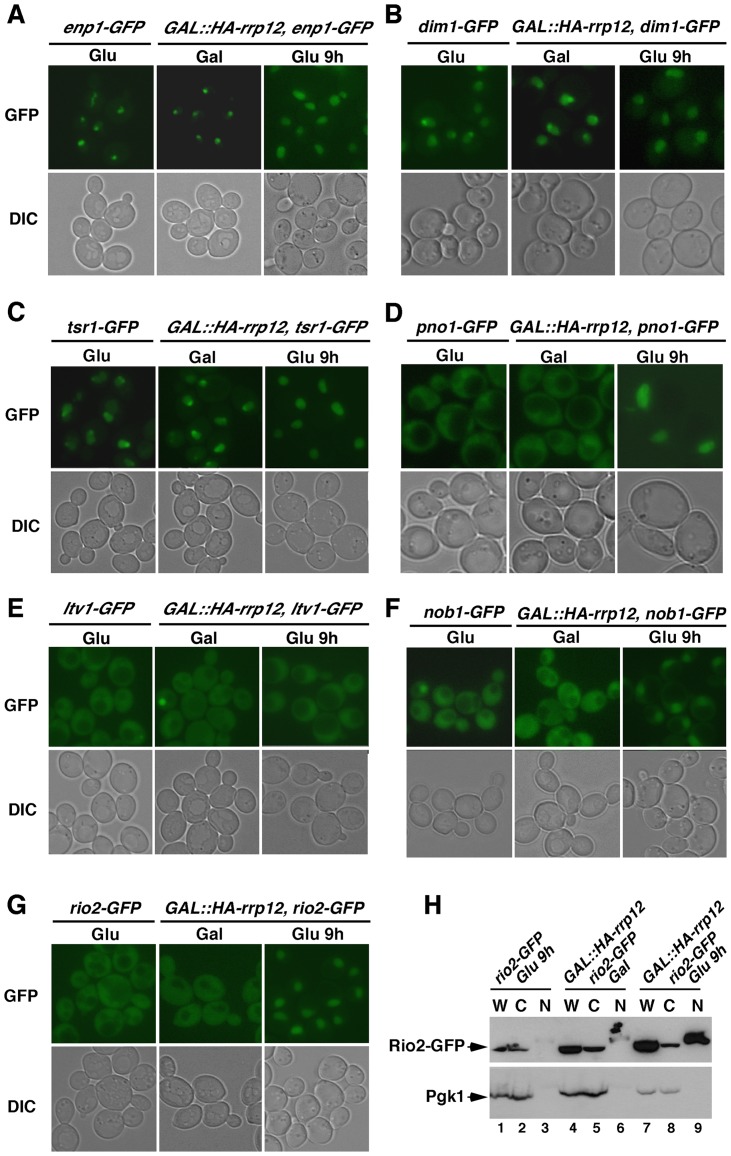
Rrp12 is required for the export of pre-40S particles out of the nucleus. (**A** to **G**) Top panels, epifluorescence microscopy analysis of the subcellular distribution of GFP-tagged Enp1 (A), Dim1 (B), Tsr1 (C), Pno1 (D), Ltv1 (E), Nob1 (F) and Rio2 (G) in the indicated yeast strains and culture conditions (top). Bottom panels, differential interference contrast (DIC) images of the above preparations. (**H**) Western blot analysis showing the distribution of Rio2-GFP (top panel) and Pgk1 (bottom panel) in whole cell lysates (W), cytosolic (C) and nuclear (F) fractions obtained from either control *rio2-GFP* cells (lanes 1 to 3) or *GAL::HA-rrp12/rio2-GFP* cells growing in galactose-containing medium (lanes 4 to 6) or upon a shift to glucose-containing medium for 9 hours (lanes 7 to 9).

The above findings indicated that the lack of Rrp12 blocks the 40S synthesis pathway at a step downstream the assembly of pre-40S particles. To investigate if this block occurred in the nucleolus, nucleoplasm or cytoplasm, we analyzed the subcellular localization of GFP-tagged versions of pre-40S particle (Enp1, Dim1, Pno1, Tsr1, Ltv1, Nob1, Rio2) and mature 40S subunit (Rps2) components in control and Rrp12-depleted cells. Consistent with previous reports [18], [28], [29], [30], [31], we found that these proteins exhibit nucleolar (Enp1, **Figure 4A**), nucleolar and nucleoplasmic (Dim1, Tsr1; **Figure 4B** and **Figure 4C**, respectively), and nucleoplasmic plus cytoplasmic (Pno1, Ltv1, Nob1, Rio2 and Rps2; **Figures 4D** to **G**, and **Figure S3**, respectively) localizations in both wild type cells and control *GAL::HA-rrp12* cells. However, in Rrp12-depleted *GAL::HA-rrp12* cells, we detected that most of those proteins undergo a major relocalization towards the nucleoplasm (**Figures 4A** to **G**; and **Figure S3**). The only exception was again Ltv1, since its subcellular distribution is fully Rrp12-independent (**Figure 4E**). The nuclear accumulation of Rio2, but not of the cytosolic Pgk1 control protein, in the absence of Rrp12 was demonstrated using independent subcellular fractionation experiments (**Figure 4H**). This effect is specific for the 40S subunit synthesis pathway, because the loss of Rrp12 does not alter the normal subcellular distribution of Rpl25 and Rpl11 (**Figure S3**), two 60S subunit components. These results show that pre-40S particles are blocked in the nucleoplasm when Rrp12 is absent. Collectively, our data indicate that Rrp12 does not participate in the major assembly events of pre-40S particles in the nucleus, and that it is essential for some event that immediately precedes or is concomitant to nuclear export.

### Rrp12 influences an intermediate maturation step within a 90S transitional particle

**Figure 5:**
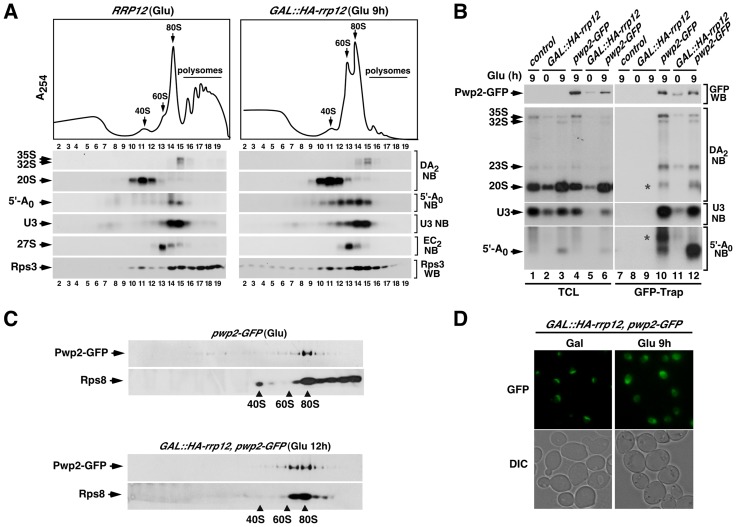
Loss of Rrp12 causes accumulation of 5′-A_0_-containing 90S pre-ribosomes. (**A**) Top panel, sucrose-gradient sedimentation analysis of ribosomal fractions (40S, 60S, 80S and polysomes) of cell lysates from the control wild type strain grown in glucose-containing media, and the *GAL::HA-rrp12* strain grown in galactose-containing media and shifted to glucose-containing media for 9 hours. Bottom panels, Northern (second to sixth panels from top) and Western (bottom panel) blot analyses of indicated components of pre-ribosomal particles in fractions obtained in the gradients. Numbers of fractions are shown at the bottom. Blotting probes and antibodies are indicated on the right. (**B**) Northern blot analysis showing copurification (second to fourth panels on the right) of the indicated pre-RNA species, U3 snoRNA and 5′-A_0_ fragment with Pwp2-GFP in the indicated yeast strains and culture conditions (top). As control, parallel Northern blots were performed on total RNAs prepared from the same total cell lysate samples used for the immunoprecipitations (second to third panels on the left). Western blot experiments were performed to analyze the amount of Pwp2-GFP present in the total cell lysates (top panel on the left) and GFP-Trap purified complexes (top panel on the right). Asterisks indicate pre-rRNA species that do not correspond to any major processing intermediate, which probably are 35S partial degradation products. (**C**) Sucrose gradient analysis showing the sedimentation behavior of Pwp2-GFP and Rps8 in the presence (top two panels) and absence (bottom two panels) of Rrp12. The positions of the gradient where 40S, 60S and 80S complexes sedimented are indicated by arrows. (**D**) Top panels, epifluorescence microscopy analysis of the subcellular distribution of Pwp2-GFP before (top left panel) and upon depletion (top right panel) of Rrp12. Bottom panels, DIC images of above preparations.

In addition to the block in pre-40S particle export, the depletion of Rrp12 causes defects in the cleavage of the pre-rRNA at site A_2_ and in the elimination of the 5′-A_0_ fragment. The accumulation of this byproduct appears to be a rather specific feature, because it is not observed upon depletion of other factors, like Pno1, that do not affect the A_0_ cleavage but are essential for the A_2_–A_3_ cleavages (**Figure S4A**). We also found that the 5′-A_0_ fragment associates to Rrp12 in wild type cells (**Figure S5**), suggesting that Rrp12 might influence directly the elimination of this fragment. As a first approximation to obtain clues about the role of Rrp12 in this process, we decided to study the sedimentation behavior of the 5′-A_0_ fragment on sucrose gradients in the presence and absence of Rrp12. These experiments corroborated the increase in the abundance of the 5′-A_0_ fragment already seen by Northern blot analyses in Rrp12-depleted cells (see above, **Figure 1D**) and, in addition, revealed that this fragment was present in complexes that sediment broadly between the 60S and 90S regions of the gradient (**Figure 5A**; right panels, gradient fractions 12 to 15). A significant proportion of these entities cosedimented with the 32S pre-rRNA and U3 snoRNA (**Figure 5A**; right panels, gradient fractions 14,15), suggesting that they form part of a 90S transitional particle that has initiated, but not completed, the processing of the 35S pre-rRNA. This interpretation is consistent with the delay in the A_2_ cleavage evidenced by the formation of aberrant 21S pre-rRNA (see above, **Figure 1D**), and the increased coimmunoprecipitation of the 5′-A_0_ fragment with the 90S pre-ribosome-specific Pwp2 protein in Rrp12-depleted cells (**Figure 5B**, compare lanes 10 and 12). The interaction of Pwp2 with the 5′-A_0_ fragment appears to take place in the context of a 90S pre-ribosome-like particle, as inferred from the presence of Pwp2 in 80–90S complexes in Rrp12-depleted cells (**Figure 5C**). In agreement with an abnormal accumulation of a 90S transitional particle, we observed by microscopy experiments that Pwp2 shifted from an exclusively nucleolar localization to a more disperse distribution between the nucleolus and the nucleoplasm upon depletion of Rrp12 (**Figure 5D**). These results indicate that the loss of Rrp12 delays some event during pre-40S particle assembly in the nucleolus, leading to both the accumulation and delocalization of 90S transitional particles in the nucleoplasm.

### The Rrp12-dependent maturation step precedes the A_2_ cleavage and the exosome-mediated degradation of the 5′-A_0_ fragment

**Figure 6:**
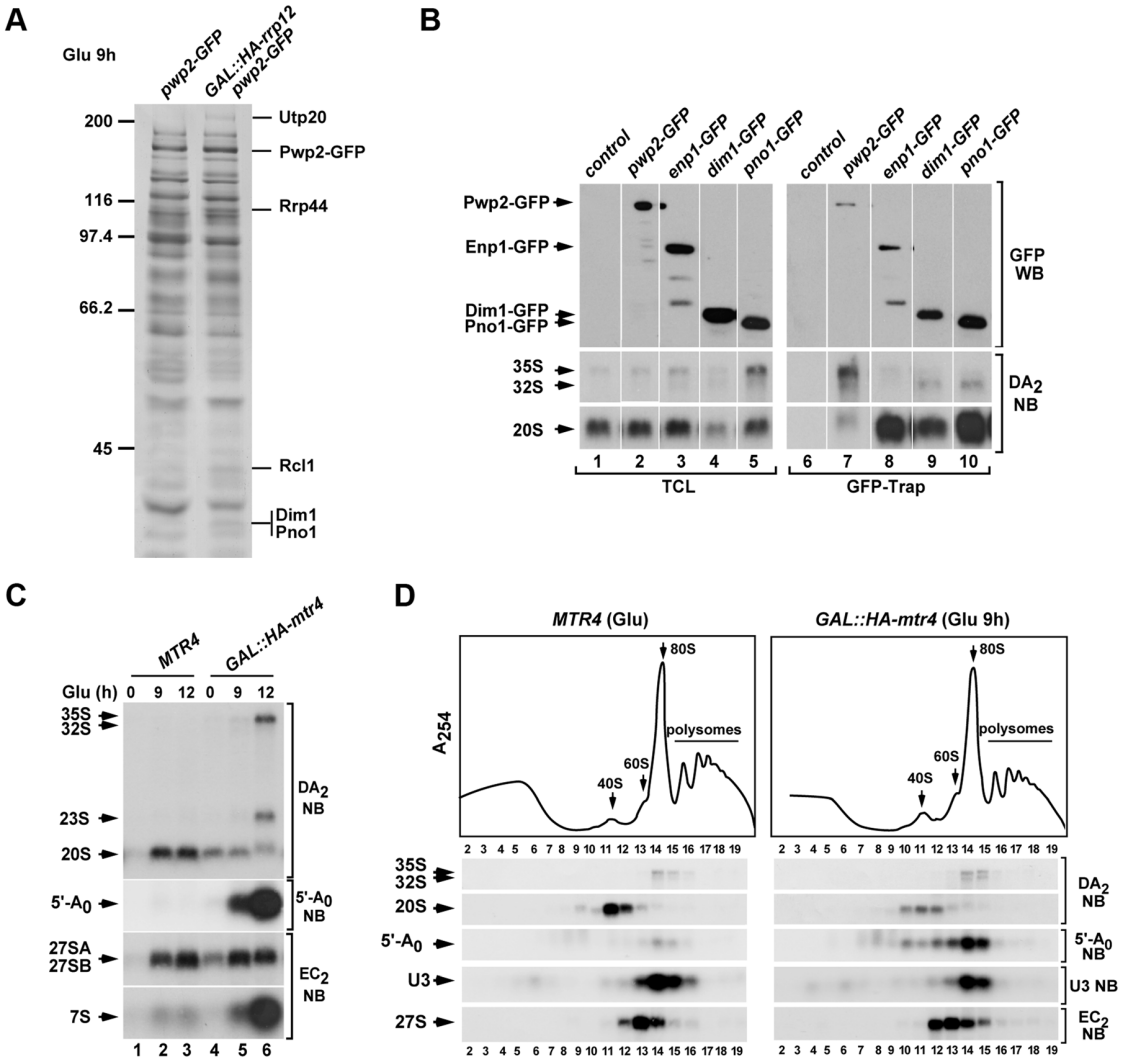
Rrp12 is required at a 90S particle-mediated maturation step that precedes exosome action. (**A**) Protein complexes formed by Pwp2-GFP in control and Rrp12-depleted cells. Bands and proteins identified by mass spectrometry are indicated on the right. Molecular weight markers (in kDa) are indicated on the left. (**B**) Northern blot analysis showing copurification (lanes 6 to 10) of the indicated pre-RNA species with GFP-tagged Pwp2, Enp1, Dim1 and Pno1 in normal cells. As control, parallel Northern blots were performed on total RNAs prepared from the same samples used for the immunoprecipitations (second and third panels on the left). Western blot experiments were performed to analyze the amount of the GFP-tagged protein present in the corresponding total cell lysates (top panel on the left) and GFP-Trap purifications (top panel on the right). (**C**) Northern blot analysis of total RNAs extracted from *MTR4*, and *GAL::HA-mtr4* cells to show the relative contents of pre-rRNA species and 5′-A_0_ fragment. Cells were grown at 30°C in galactose-containing media and shifted to glucose-containing media for the indicated times. Northern blot probes are indicated on the right. (**D**) Top panel, sucrose-gradient sedimentation analysis of ribosomal fractions (40S, 60S, 80S and polysomes) of cell lysates from control (*MTR4*) and Mtr4-depleted (*GAL::HA-mtr4* (Glu 9 h)) strains. Bottom panels, Northern blot analysis of indicated components of pre-ribosomal particles in gradient fractions obtained in the above experiment. Numbers of fractions are shown at the bottom. Blotting probes and antibodies are indicated on the right.

We next characterized by mass spectrometry the complexes formed by Pwp2 in the absence of Rrp12 to investigate possible differences in the composition of 90S pre-ribosomes. Although highly similar to those formed in control cells, we observed the presence of new Pwp2 partners in the absence of Rrp12 (**Figure 6A**). Those included 90S pre-ribosome components involved in the cleavage of the 35S precursor at the A_0_–A_1_–A_2_ (Utp20, Rcl1) and A_1_–A_2_ (Dim1, Pno1) sites [29], [32], [33], [34], [35], [36]. Interestingly, we observed using RNA coimmunoprecipitation experiments that two of the above partners, Dim1 and Pno1, preferentially bind the 32S rather than the earliest 35S pre-rRNA (**Figure 6B**). This suggests that they become stably assembled onto the 90S pre-ribosome upon cleavage of the 35S precursor at the A_0_ and A_1_ sites (see above, **Figure 1A**). We also found among the new partners the nuclease Rrp44 (also known as Dis3), an exosome subunit shown to be involved in the direct physical interaction with the 5′-A_0_ fragment [37]. This finding was quite interesting for us, because previous results have shown that this interaction seems to be crucial for poising the 5′-A_0_ fragment for productive degradation [37], [38]. Thus, we surmised that the Rrp44-Pwp2 interaction detected in Rrp12-depleted cells could indicate that the exosome is normally recruited to 90S pre-ribosomes and that, in the absence of Rrp12, there is an enrichment or stabilization of some of those exosome-containing 90S pre-ribosomes. In agreement with this idea, we found using sucrose gradient sedimentation analyses that Rrp44 is indeed present in 80–90S complexes both in control and Rrp12-depleted cells (**Fig. S1D**). These data raised the possibility that the defect in the elimination of 5′-A_0_ fragment found in Rrp12-depleted cells could be due to an impairment of exosome function. Consistent with this idea, we found that the elimination of the exosome cofactor Mtr4 (also known as Dob1) elicited the expected accumulation of the 5′-A_0_ fragment (**Figure 6C** and **Figure 6D**) [39] and, most importantly, that such accumulation occurs in the context of 80–90S complexes, similarly to what is observed in Rrp12-depleted cells (**Figure 6D**; see above, **Figure 5**). Interestingly, Rrp12-depleted cells do not exhibit the sustained high levels of the 5′-A_0_ fragment seen in Mtr4-depleted cells (**Figure 1D** and **Figure 6C**), indicating that the exosome activity is affected but not fully compromised upon the loss of Rrp12. Consistent with this, we have seen that the loss of this protein does not trigger other terminal defects typically observed in exosome-deficient cells, such as the abnormal accumulation of the 35S pre-rRNA, the total block of 7S pre-rRNA maturation, and the balanced decrease in the contents of both ribosomal subunits (**Figure 6C** and **Figure 6D**) [39], [40]. Taken together, our data indicate that the loss of Rrp12 causes a 90S pre-ribosome maturation defect that precedes the A_2_ cleavage and the exosome-dependent 5′-A_0_ fragment degradation steps. As a result, it promotes either a delay or partial inhibition, but not a block, in the A_2_ cleavage of the pre-rRNA and the elimination of the 5′-A_0_ fragment.

### The Crm1 exportin is also involved in the Rrp12-dependent 90S pre-ribosome maturation step

**Figure 7:**
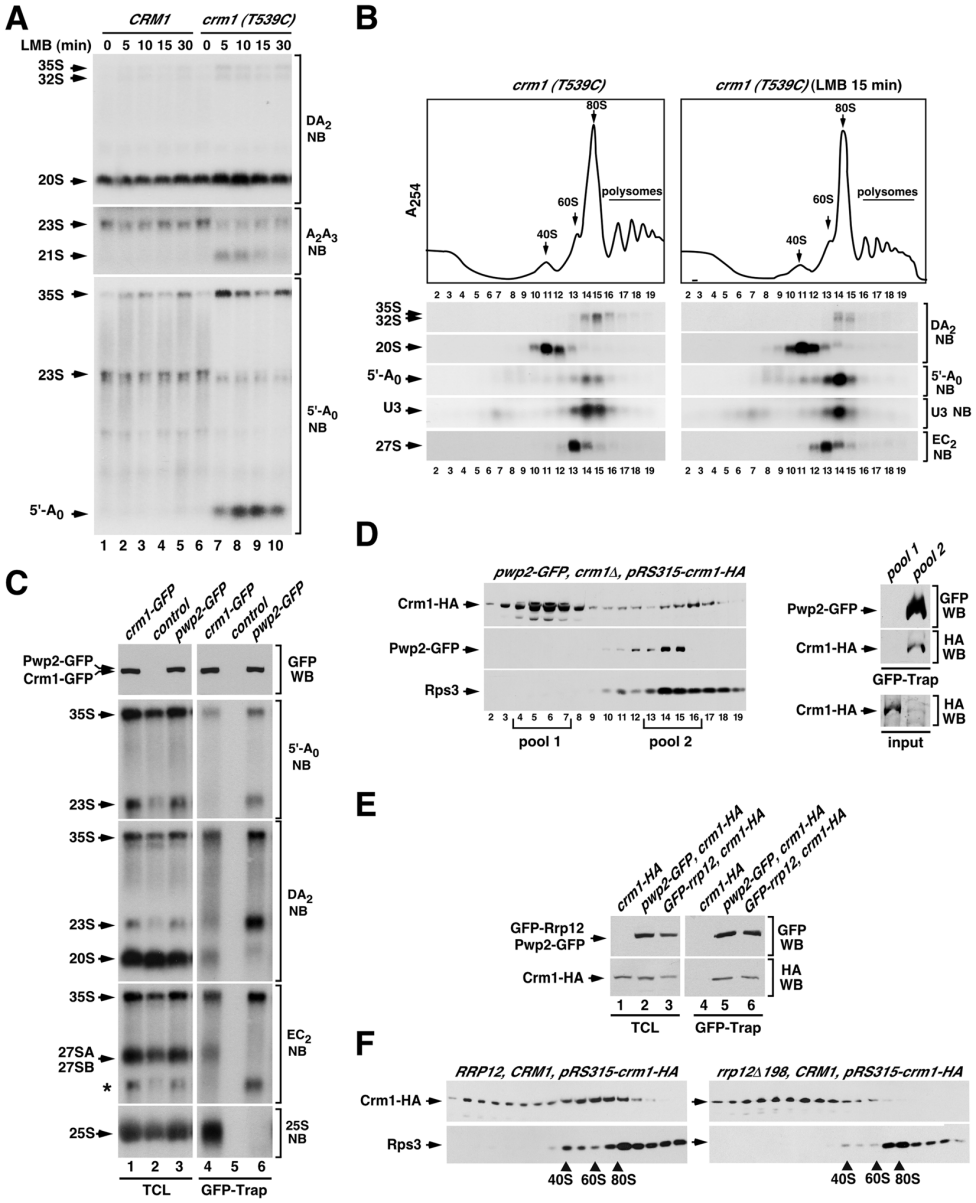
Crm1 participates in the Rrp12-mediated 90S maturation step. (**A**) Northern blot analysis showing the amount of the indicated pre-RNA intermediaries and 5′-A_0_ byproduct (left) in control *CRM1* and mutant *crm1 (T539C)* strains treated with leptomycin B (LMB) for the indicated periods of time (top). (**B**) Top panel, sucrose-gradient sedimentation analysis of ribosomal complexes (40S, 60S, 80S and polysomes) of cell lysates from *crm1 (T539C)* cells that were either nontreated (left panels) or treated (right panels) with leptomycin B for 15 min. Bottom panels, Northern blot analysis of the indicated pre-rRNA species in the gradient fractions. Numbers of fractions are shown at the bottom. Blotting probes are indicated on the right. (**C**) Northern blot analysis showing copurification (second to fifth panels on the right) of the indicated pre-RNA species with GFP-tagged Crm1 (lane 4) and GFP-tagged Pwp2 (lane 6) in normal cells. Control samples were wild-type cells expressing endogenous untagged Crm1 and Rrp12. Parallel Northern blots were performed on total RNAs prepared from the same total cell lysate samples used for the purifications (second to fifth panels on the left). Western blot experiments were performed to analyze the amount of Crm1-GFP and Pwp2-GFP present in the total cell lysates (top panel on the left) and GFP-Trap purified complexes (top panel on the right). The asterisk in the EC_2_ blot indicates the signal of the 23S pre-rRNA from previous hybridization with the DA_2_ probe. (**D**) Sucrose gradient analysis of Crm1-HA, Pwp2-GFP and Rps3 in *pwp2-GFP*/*crm1Δ* cells containing a pRS315-crm1-HA plasmid. Gradient fractions were analyzed by Western blot with anti-HA, anti-GFP and anti-Rps3 (left three panels). The right panels show copurification of Crm1-HA with GFP-Trap purified complexes from pooled fractions of the 80–90S gradient region (pool 2). A GFP-Trap purification on pooled fractions of the 10–20S gradient region (pool 1) was used as a control. Parallel Western blots analyzed the amount of Crm1-HA in each one of the pool samples used for the GFP-Trap purifications (input) (right bottom panel). (**E**) Western blot analysis showing copurification of Crm1-HA with GFP-tagged Pwp2 (lane 5) and with GFP-tagged Rrp12 (lane 6) in *pwp2-GFP* cells containing a pRS315-crm1-HA plasmid (lane 5), and in *GFP-rrp12* cells containing a pRS315-crm1-HA plasmid (lane 6), respectively. Parallel Western blots were performed to analyze the amounts of the coimmunoprecipitated proteins in total cell lysates (lanes 1 to 3). (**F**) Sucrose gradient analysis showing the sedimentation behavior of Crm1-HA and Rps3 in *RRP12/CRM1* (left panels) and *rrp12-Δ198/CRM1* (right panels) cells containing a pRS315-crm1-HA plasmid. The positions of the gradient where 40S, 60S and 80S complexes sedimented are indicated by arrows.

Given the implication of Rrp12 in the export of pre-40S particles (see above, **Figure 3** and **Figure 4**), we decided to investigate whether the pre-40S export step was associated to the Rrp12-dependent 90S pre-ribosome maturation step. If that were the case, we expected that the elimination of any other protein involved in pre-40S export would induce the same defects seen in Rrp12-depleted cells. To test this idea, we chose a yeast strain that constitutively expressed a mutant version of the Crm1 (Crm1^T539C^) exportin. This mutant protein, unlike its wild type counterpart, can be specifically inhibited by leptomycin B [41]. Using this strategy, we found that the inhibition of Crm1 recapitulates all the defects observed in Rrp12-depleted cells, including increased abundance of the 35S, 32S and 21S pre-RNA species (**Figure 7A**), abnormal levels of the 5′-A_0_ fragment (**Figure 7A** and **Figure 7B**), accumulation of this fragment in 80–90S complexes (**Figure 7B**) and, as expected [19], an increase in the content of the 20S pre-rRNA due to the halt in pre-40S particle nuclear export (**Figure 7A**). These results indicate that the 40S subunit export machinery facilitates a late 90S pre-ribosome maturation event that promotes the rapid cleavage of the pre-rRNA at site A_2_ and the efficient degradation of the 5′-A_0_ fragment. This function is quite specific for export regulators, because the elimination of factors specifically involved in the cytoplasmic maturation of pre-40S complexes (Rio2 and Ltv1) does not trigger any of the above defects [16], [28], [42] (**Figure S4B**).

The above results led us to investigate whether Crm1, like Rrp12, was present in 90S pre-ribosomes. We first assessed the potential interaction of Crm1 with two 90S pre-ribosome components, the 35S pre-RNA and Pwp2, using coimmunoprecipitation analyses similar to those that detect Rrp12 in 90S and pre-40S particles (see above Figure 2). This approach however did not reveal associations of Crm1 with any pre-ribosomal component, not even with pre-rRNAs or proteins present in the pre-40S and pre-60S complexes transported by this exportin. We therefore decided to change the experimental conditions of our coimmunoprecipitation assays. In particular, we changed the Triton X-100-containing lysis buffer by a NP-40-containing buffer that was similar to buffers used by others to detect interactors of Crm1 in vivo [43], [44], [45]. Notably, when we purified Crm1-GFP using the NP-40 buffer, we could readily observe that it interacts with the 35S pre-rRNA, the 20S pre-rRNA, 27S pre-rRNAs and the 25S rRNA (Figure 7C). The associations with these RNAs were specific because in the same Northern blots Pwp2-GFP coprecipitated the 35S and 23S pre-rRNAs, but not the 20S, 27S and 25S RNAs. These results indicate that Crm1 binds to pre-40S and pre-60S particles, as expected from its role in export, and also that it is already recruited to early 90S particles. Consistent with this, we found using sucrose gradient sedimentation analysis that Crm1 is indeed present in large 80–90S complexes that co-sediment with Pwp2 (Figure 7D). Furthermore, when 90S pre-ribosomes were purified from sucrose gradients using Pwp2 as bait it was confirmed that they do contain Crm1 (Figure 7D, right set of panels). Western blot analysis of Rrp12-containing complexes from total cell lysates evidenced that Crm1 interacts with Rrp12 (Figure 7E), a result consistent with the common presence of the two proteins in both 90S and pre-40S pre-ribosomes.

In our final set of experiments, we investigated whether the recruitment of Crm1 to 90S pre-ribosomes was Rrp12-dependent. For this purpose we analyzed the sedimentation behavior in sucrose gradients of a HA-tagged version of Crm1 that was coexpressed with the endogenous Crm1 either in wild type or in *rrp12Δ198* cells. We found that in wild type cells the Crm1-HA protein is recruited to large assemblies, including 80–90S-like complexes (**Figure 7F**, left two panels). This sedimentation in large complexes is drastically reduced in *rrp12Δ198* cells (**Figure 7F**, right two panels), suggesting that the incorporation of Crm1 onto large 80–90S pre-ribosomal particles is Rrp12-dependent. Altogether, our data indicate that Rrp12 and Crm1 act on 90S pre-ribosomes in a concerted manner.

## Discussion

**Figure 8:**
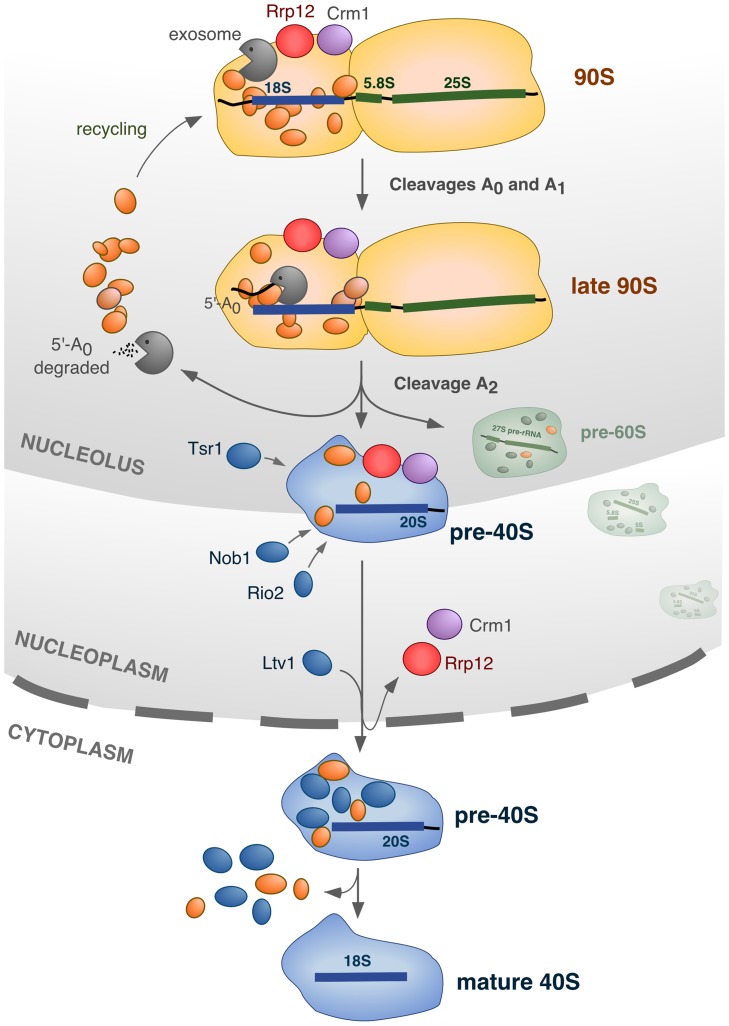
Model for the integration of different processes in the nucleolus during synthesis of 40S subunits. The 90S pre-ribosome contains ∼70 factors (represented in orange) that are specifically required for the cleavage of the primary pre-rRNA at sites A_0_, A_1_ and A_2_, and for the assembly of ribosomal proteins (not represented). In addition, the 90S pre-ribosome engages two other sets of proteins that participate in activities that will be initiated at the time of, or immediately after, the A_2_ cleavage: the exosome complex, and Rrp12/Crm1. The exosome degrades the 5′-A_0_ fragment, allowing the liberation and recycling of bound 90S proteins. Rrp12 and Crm1 act as export factors for the released pre-40S particle. The cleavage of the pre-rRNA at site A_2_ is intertwined with the initiation of 5′-A_0_ degradation and the priming of the emergent pre-40S particle for nuclear export. During the rapid transit of the pre-40S particle from the nucleolus to the cytoplasm, a few maturation factors (Tsr1, Rio2, Nob1) that will be required in the cytoplasm are incorporated in a manner independent of nuclear export. Another maturation factor, Ltv1, requires Rrp12 for its stable incorporation onto pre-40S particles, but whether or not it is dependent on the export process itself remains to be ascertained. Further details about this model, and the evidence supporting it, is given in the text.

The results presented here identify Rrp12 as a factor required for a number of intertwined steps of the 40S ribosomal subunit synthesis pathway (**Figure 8**). We have observed that Rrp12, together with Crm1, is first recruited to the pathway to facilitate the processing of the 35S pre-rRNA and the elimination of the 5′-A_0_ fragment in the context of a late 90S transitional particle (**Figure 8**). A lack of Rrp12 or Crm1 at this step delays but does not halt the assembly and release of early pre-40S particles. Interestingly, this early function of Rrp12 occurs immediately upstream and temporally close to the export of the pre-40S particles, a process that absolutely requires Rrp12 and Crm1. In addition to revealing a hitherto unknown role for export-related factors in a specific maturation step in the nucleolus, our results shed light onto the dynamics of 90S pre-ribosome factors upon cleavage of the 35S pre-rRNA at site A_2_. Indeed, some authors previously suggested that, after the A_2_ cleavage, the non-ribosomal components of the 90S particle are released en bloc in association with the 5′-A_0_ fragment [3], [18]. However, the formation of such disassembly complexes, and when and how was the exosome recruited, remained unclear. We find no evidence for the formation of a post-disassembly complex containing the 5′-A_0_ fragment upon which the exosome acts (**Figure 6D**). Rather, our results indicate that the exosome is present in transitional 90S pre-ribosomes to degrade the 5′-A_0_ fragment, either in the last step of pre-40S particle assembly or at the very time of pre-40S particle release (**Figure 8**). The implication of Crm1 in steps of ribosome synthesis, other than nuclear export, is also a new finding in yeast. In human cells, Crm1 has been implicated in the targeting of snoRNP complexes to the nucleolus [43], [46]. Whether Rrp12 and Crm1 utilize the same domains for the export-related and maturation-related functions, and whether the two proteins need to interact directly to exert their functions, remains to be determined. We have found that Rrp12 and Crm1 purified from bacteria do not stably interact *in vitro* (unpublished data). However, we cannot exclude the possibility that such interaction could require the participation of other proteins. Indeed, it has been shown before that the interaction of Crm1 with other molecules involves the participation of additional factors, including the Ran GTPase in its GTP-bound state. Ran can in fact be involved in these interactions, as suggested by the identification of allele-specific Ran mutants that elicit defects in the degradation of the 5′-A_0_ fragment [47]. Based on the present results, we hypothesize that such defects could be associated to the Rrp12- and Crm1-dependent mechanism reported here. An involvement of Ran on the association of Crm1 with pre-ribosomes could also explain the difficulties for detecting Crm1 in purified 90S and pre-40S pre-ribosomes, because these complexes are normally prepared under conditions that favor the conversion of Ran-GTP to Ran-GDP. Here we describe that, using a buffer that contains 0.2% NP-40, it is possible to detect the specific association of Crm1 to both pre-rRNAs and pre-ribosomal components by coimmunoprecipitation analysis. The reason for the efficiency of this buffer is unclear, but it must somehow favor the maintenance of some Ran-GTP levels and/or affect other currently unknown features that improve the stability or solubilization of Crm1-containing complexes.

In our model we propose that Rrp12 is an export factor rather than a nuclear maturation factor (**Figure 8**). Consistent with this, we have observed that the elimination of Rrp12 leads to the accumulation of pre-40S particles that, in addition to being dissociated from the 90S pre-ribosome machinery, are fully-assembled. This is evidenced by the recruitment to those particles of factors that are predominantly cytoplasmic in normal cells (Rio2, Nob1), and that therefore must join the pathway just before nuclear exit. One important inference of our results is that the major assembly events involved in the formation of pre-40S particles are separable and fully independent from the subsequent export step. A direct participation of Rrp12 in the export process is also supported by the previously-described interactions of this protein with some nucleoporins and with Ran [22]. Unexpectedly, we could not find any significant role for Rrp12 in the export of pre-60S subunits, as it had been previously published [22]. In addition to the phenotypic analysis of Rrp12-depleted cells, the prominent role of Rrp12 in the 40S rather than the 60S subunit pathway is supported by the RNA-protein interaction data showing the specific binding of Rrp12 to the 20S but not the 27S and 7S pre-rRNAs. The reason for these different results is not readily apparent. We have found that the loss of Rrp12 elicits the 40S subunit-specific phenotype both in the W303 and in BY4743 strains, indicating no influence of the genetic background. Still, it is worth noting that the depletion of Rrp12 causes delays in the processing of 5.8S rRNA precursors in the nucleus by a hitherto unknown mechanism. According to our results, such delays do not impact the overall production of 60S subunits, but it could be possible that, under some experimental conditions or in strains with genetic modifications that subtly affect ribosome biogenesis, the defect in 5.8S rRNA production became exacerbated and caused nuclear accumulation of pre-60S particles. It is also plausible that Rrp12 could interact either weakly or very transiently with some pre-60S particle subpools, as it would be expected if its influence on the processing of 5.8S precursors were direct. This possibility would be in agreement with the previously-reported detection of Rrp12 bound to 27SB pre-rRNAs using primer-extension analyses [22]. Despite the possibility of these alternative scenarios, we believe that our data clearly indicate that Rrp12 is not essential for 60S subunit synthesis. Consistent with this idea, it is also worth noting that mammalian Rrp12 has been shown to be required exclusively for 40S subunit synthesis [48], [49].

One distinctive feature of the intermediate particle formed in the absence of Rrp12 is the lack of Ltv1, a factor not essential for nuclear export. Previous studies indicate that this protein is recruited in the nucleus [31], [50], but some evidence suggests that its interaction with the nuclear pre-ribosomes that are about to be exported might be weak [20]. Thus, a possible explanation for the absence of Ltv1 in the pre-40S particles of Rrp12-depleted cells is that those particles are ready to be exported and have Ltv1 loosely associated. Alternatively, it is possible that Rrp12 could be actively required for the docking of Ltv1 to those particles during the export process. We currently favor the latter possibility, since we have observed that the interaction of these two proteins can occur in a pre-rRNA-independent manner. Based on the present data, we believe that Rrp12 probably promotes the recruitment of Ltv1 onto the pre-40S particle immediately prior to the step of transport (**Figure 8**). Upon this docking step, Rrp12 is carried along with the particle through the nuclear pores to be finally released when the particles reach the cytosol. Consistent with this hypothesis, our co-purification experiments and other proteomic analyses have shown that Rrp12 is not a major component of cytoplasmic pre-40S particles. Alternatively, it is also possible that Rrp12 could remain associated to cytoplasmic pre-40S particles and only becomes released upon completion of a specific maturation event that takes place right after the nuclear export step. This model would explain previous results indicating that Rrp12 can associate with di-methylated 20S pre-rRNA, a modified form of the 20S pre-rRNA that is generated in the cytoplasm [22]. Further work will be required to dissect the fate and specific roles of Rrp12 in these late maturation stages.

The reason for using the pre-40S export machinery to facilitate late 90S pre-ribosome-mediated processes is unknown. We propose that such mechanism could ensure a timely coordination of the recycling kinetics of 90S pre-ribosome components with pre-40S particle release and rapid export (**Figure 8**). An inter-relation between these three processes is indicated by our data, which shows that the impairment of nuclear export causes defects in the function, disassembly and subcellular localization of the 90S pre-ribosome. Future work will be needed to explain the precise mechanisms by which the export factors influence the activities of the exosome and A_2_ cleavage complexes within the 90S pre-ribosome.

## Materials and Methods

### Yeast strains, genetic methods and plasmids

The *Saccharomyces cerevisiae* strains and plasmids used in this study are listed in Tables S1 and S2, respectively. The conditional strain for *RRP12* under the control of the *GAL1* promoter (YPM7) was generated by one-step insertion of a KAN-MX6-GAL1 cassette upstream of the ATG of the *RRP12* gene [51]. This strain (referred to in the text as *GAL::HA-rrp12*), and the other *GAL1*-driven strains used in this study, JDY144, WDG72, YGM168, YO470 and YGM174 (referred to in the text as *GAL::HA-spb4*, *GAL::rsa4*, *GAL::HA-pno1*, *GAL::rio2-ProtA* and *GAL::HA-mtr4*, respectively) were cultured at 30°C in media containing galactose (YPGal, 0.4% yeast extract, 0.8% peptone, 0.1 mM adenine, 2% galactose) or glucose (YPD, 0.4% yeast extract, 0.8% peptone, 0.1 mM adenine, 2% glucose). For protein depletion, the incubation times in YPD varied from 9 to 18 h, as indicated in figure labelings. The *ltv1Δ* strain was cultured at 25°C, the temperature at which the 40S subunit biogenesis defects of this strain are more exacerbated. For the experiments of inactivation of Crm1 we employed a strain with the *CRM1* gene depleted that carried a plasmid for the expression of the *crm1-T539C-HA* allele (strain MNY8, plasmid pDC-crm1-T539C). As a control for those experiments, we employed the corresponding strain carrying a plasmid for the expression of *crm1*-HA (strain MNY7, plasmid pDC-CRM1). MNY7 and MNY8 cells were treated with 100 ng/ml of leptomycin B (LMB) for 5–15 min. All strains with MYC, hemagglutinin (HA) or green fluorescent protein (GFP) carboxy-terminal tagged alleles, except the *crm1-HA* and *GFP-rrp12* ones, were generated by in-frame one-step integration of PCR cassettes in the corresponding locus of wild type cells. In these strains, the epitope-tagged versions are the only source of the proteins in the cell, and their expression is driven from the endogenous gene promoters. All epitope-tagged alleles were fully functional, as measured by normal growth rates and normal contents of rRNAs, pre-rRNAs and ribosomal subunits. The sedimentation analysis of Crm1-HA shown in Figure 7D was performed on the YGM193 strain (referred to in the figure as *pwp2-GFP*, *crm1Δ*, pRS315-crm1-HA). The coimmunoprecipitation experiment in Figure 7E was performed with the YMD6 strain carrying the pDC-CRM1 plasmid (referred to in the figure as *pwp2-GFP*, *crm1-HA*) and with the YPM7 strain carrying the pGM58 and pDC-CRM1 plasmids (referred to in the figure as *GFP-rrp12*, *crm1-HA*). The sedimentation analysis of Crm1-HA shown in Figure 7F was performed on the following strains maintained in glucose-containing media: YPM7 carrying the pBN18 and pDC-CRM1 plasmids (referred in the figure as *RRP12*, *CRM1*, *pRS315-crm1-HA*), and YPM7 carrying the pBN19 and pDC-CRM1 plasmids (referred in the figure as *rrp12Δ198*, *CRM1*, *pRS315-crm1-HA*). Preparation of media, yeast transformation and genetic manipulations were performed according to established procedures.

### RNA preparation and northern blot analysis

RNAs from total cellular lysates, gradient fractions and coimmunoprecipitations were prepared by the hot-phenol method [52]. Oligonucleotide labeling, RNA separation, Northern blotting and hybridization were performed as described previously [53]. The sequences of the oligonucleotides used as probes are shown in Table S3.

### Protein purification and analysis

Preparation of total celular lysates for immunoblot, Western blot analysis, purification of GFP-tagged proteins and mass spectrometry analysis were performed as described previously [23], except for the Pwp2-GFP/Crm1-HA and GFP-Rrp12/Crm1-HA coimmunoprecipitation analysis in Figure 7E. In this case, instead of lysing cells in IP buffer (20 mM Tris-HCl, pH 7.5, 5 mM MgCl_2_, 150 mM potassium acetate, 1 mM dithithreitol, 0.2% Triton X-100, supplemented with Complete [Roche]), cells were lysed in IP-NP40 buffer (15 mM Na_2_HPO_4_, 10 mM NaH_2_PO_4_, pH 7.2, 150 mM NaCl, 2 mM EDTA, 50 mM NaF, 0.1 mM NaVO_4_, 0.5% NP-40 Alternative [Calbiochem], supplemented with Complete). Before purification of Pwp2-GFP and Rrp12-GFP with GFP-TRAP (Chromotek), the pre-cleared lysates were diluted to 0.2% NP-40. The anti-Rrp12 antibody used for Western blot in Figure 2B is a rabbit polyclonal antibody raised against a peptide mapping at the C-terminus of yeast Rrp12 (this study). Other antibodies used in Western blot analysis were: anti-MYC (Roche), anti-GFP (Clontech), anti-HA (Covance), anti-Nop1 (Pierce), anti-Mex67 (kind gift of C. Dargemont, Institut Jacques Monod), anti-Rps3 (kind gift of M. Seedorf, University of Heidelberg), anti-Rps8 (kind gift of G. Dieci, University of Parma), anti-Rpl1 (kind gift of F. Lacroute, Centre de Génétique Moléculaire, Gif-sur-Yvette), anti-Pgk1 (Abcam), and anti-Cdc11 (Santa Cruz). For the represention of the results of the proteomic analysis shown in Figure 3E, the four different dot sizes are indicative of the amount of the copurifying protein relative to the amount of bait: >80%, 60–80%, 40–60%, and <40%.

### Polysome preparation and sucrose gradient analysis

Cell cultures (200 ml) were grown to an optical density at 600 (OD_600_) between 0.8 and 0.1 and, before harvesting, cycloheximide was added to a final concentration of 0.1 mg/ml. After an incubation on ice for 5 min, cells were collected and lysed in 700 µl of HK buffer (20 mM HEPES, pH 7.5, 10 mM KCl, 2.5 mM MgCl_2_, 1 mM EGTA, 1 mM dithiothreitol (DTT) and 0.1 mg/ml cycloheximide) using a Fastprep apparatus. Cell lysates were pre-cleared by high-speed centrifugation, and extracts equivalent to 5–20 absorption units at 260 nm (A_260_) were loaded on 7–50% sucrose gradients (10 ml), which had been prepared in HK buffer without cycloheximide. Ultracentrifugation, subsequent fraction collection and polysome profile recording were performed as previously described [53]. For Western blot analysis, 40 µl samples of each fraction were mixed directly with 10 µl of SDS-PAGE loading buffer (SPLB) and loaded onto SDS polyacrylamide gels. For Northern blot analysis, total RNA was prepared by the hot-phenol procedure from 100 µl samples of each fraction and separated on 1.2% agarose-formadehyde gels. For the analysis of purified complexes shown in Figure 7D, two sets (pools 1 and 2) of four combined fractions were concentrated 6-fold by spinning on Microcon-10 (Millipore) filters. The recovery of proteins after the concentration step was ∼10 fold more efficient for pool 1 than for pool 2, probably due to the higher sucrose concentration in pool 2. Before performing the GFP-Trap purification, each concentrated pool was taken to 1 ml with NP-40 buffer (0,2% final concentration).

### Protein-RNA coimmunoprecipitation experiments

Cell cultures were grown to OD_600_ between 0.8 and 1.0, and polysome extracts were prepared as described above. Extract equivalents to 15 A_260_ units were taken to 250 µl with HK buffer and mixed with 0.5 ml of IP buffer containing Complete and 600 U/ml of RNasin (Promega). In the Crm1-RNA coimmunoprecipitations shown in Figure 7C, instead of using IP buffer it was used IP-NP40 (0.2% final concentration) buffer. For evaluation of protein content in total cell lysates, a 30 µl aliquot of the pre-cleared lysate was mixed with 30 µl of SPLB and kept frozen until analysis by Western blot. The rest of the extract was incubated with 2 µg of anti-MYC 9E10 (Roche) antibody or with 25 µl of GFP-TRAP beads at 4°C for 2 h. When using anti-MYC antibody, immunoprecipitates were immobilized with GammaBind sepharose beads (GE Healthcare). Immunoprecipitates were washed four times at 4°C with IP or IP-NP40 buffer. For protein analyses, one fifth of the immunoprecipitated material was resuspended in SPLB and analyzed, in paralel with the samples of total protein, by Western blot. For RNA analyses, the rest of the immunoprecipitated material was resuspended in 400 µl of 50 mM sodium acetate, 10 mM EDTA (pH 5.2), and processed for RNA extraction by the hot phenol method. After ethanol precipitation, the whole amount of recovered RNA was resuspended in formaldehyde loading buffer, separated on 1.2% agarose-formadehyde gels and analyzed by Northern blot. In parallel, in the same Northern blot experiments, it was evaluated the pre-rRNA content in cell lysates before immunoprecipitation, using 5 µg of total RNA prepared by the hot phenol method directly from extract equivalents to 10 A_260_ units of the corresponding polysome preparations.

### Fluorescence microscopy

Cells were visualized using a Zeiss Axioplan 2 microscope equiped with a 63× objective, a Hammamutsu ORCA-ER digital camera and Openlab (Improvision) cell imaging analysis software. The Rpl25-EGFP and Rps2-GFP reporter assays to monitor pre-40 and pre-60S nuclear accumulation were performed as previously described [54].

### Subcellular fractionation

Cells were grown to OD_600_ between 0.8 and 0.1, harvested and spheroplasts prepared by incubation in S buffer (50 mM Tris-HCl, pH 7.5, 10 mM MgCl_2_, 1.2 M sorbitol, 1 mM dithiothreitol, 5 mg/ml Zymolyase T-100 (Seikagaku) at 30°C for 15 min. After two washes with the same buffer, the spheroplasts were lysed using a manual homogenizer in Ficoll buffer (10 mM Tris-HCl, pH 7.5, 20 mM KCl, 5 mM MgCl_2_, 3 mM dithiothreitol, 1 mM EDTA, 1 mM PMSF, 180 mg/ml Ficoll-400, supplemented with Complete). Pre-cleared lysates were ultracentrifuged in a TLA 100.3 rotor at 23.000 rpm for 15 min, and the supernatant cytosolic fraction collected. The nuclei pellet was resuspended in 50 mM Tris-HCl, pH 7.5, 100 mM NaCl, 30 mM MgCl_2_, 0.25% NP-40 supplemented with Complete. Aliquots of the precleared whole lysate (W), cytosolic fraction (C) and nuclei (N) were mixed with SPLB and loaded onto a SDS polyacrilamide gel for Western blot analysis.

## Supporting Information

Figure S1 Recruitment of maturation factors to pre-40S particles in the absence of Rrp12. (A–C) Sucrose gradient analysis showing the sedimentation behavior of Ltv1-MYC (A), Enp1-MYC (B), Rio2-MYC (C) and Rrp44-GFP (D) in the presence (top two panels) and absence of Rrp12 (bottom two panels). Each set of gradient fractions was analyzed by Western blot with anti-MYC (A, B and C) of anti-GFP (D), and anti-Rps3. The positions of the gradient where 40S, 60S and 80S complexes sedimented are indicated by arrows.
(TIF)

Figure S2 Rrp12 is not required for the association of Prp43 and Mex67 with pre-40S particles. (**A**) Northern blot analysis showing coimmunoprecipitation of the indicated pre-RNA species with Prp43-MYC in the presence and absence of Rrp12. Total RNAs (middle and bottom panels, lanes 1 to 6) and RNAs present in Prp43-MYC immunoprecipitates (middle and bottom panels, lanes 7 to 12) obtained from the indicated strains, grown under the indicated conditions, were analyzed with a probe that maps to the pre-rRNA D-A_2_ region. Western blot experiments were performed to analyze the amount of Prp43-MYC present in the total cell lysates (top panels, lanes 1 to 6) and immunoprecipitations (top panels, lanes 7 to 12). (**B**) Western blot analysis showing copurification of Mex67 with Tsr1-GFP in the presence and absence of Rrp12. Total cell lysates (lanes 1 to 6) and GFP-Trap purified complexes (lanes 7 to 12) obtained from the indicated yeast strains, grown under the indicated conditions, were analyzed with anti-MYC and anti-Mex67 antibodies. The thin white lines between lanes 3 and 4, and lanes 9 and 10, shown in A and B, indicate the presence of in-between lanes in the same blot that have been removed.
(TIF)

Figure S3 The loss of Rrp12 causes accumulation of pre-40S, but not pre-60S, complexes in the nucleus. Epifluorescence microscopy analysis of *GAL::HA-rrp12* cells (A, C), control *GAL::HA-spb4* cells (B), and control *GAL::HA-rsa4* cells (D) expressing 40S (Rps2-GFP; top and second panels in A and B), 60S (Rpl25-GFP, third and bottom panels in A and B; and Rpl11-GFP, top and bottom panels in C and D) subunit reporters. These cells were grown in galactose-containing medium or shifted to glucose-containing medium for 18 h as indicated. The GFP signal, the DAPI-stained nuclei and the GFP-DAPI merge are shown in the left, middle and right panels, respectively.
(TIF)

Figure S4 The loss of Pno1, Rio2 or Ltv1 does not cause accumulation of the 5′-A_0_ fragment. Northern blot analysis of total RNAs extracted from *GAL::HA-rrp12* and *GAL::HA-pno1* cells (A), and from *GAL::HA-rrp12*, *GAL::rio2* and *ltv1Δ* cells (B). Cells were grown at 30°C (except those corresponding to the lanes marked with an asterisk in B) in galactose-containing media or shifted to glucose-containing media for the indicated times. The samples marked with an asterisk (lanes 10 and 11 in B) were prepared from cultures grown at 25°C, the temperature at which the defects of the *LTV1* deletion are most patent. The specific region of the 35S pre-rRNA recognized by each Northern blot probe is indicated on the right.
(TIF)

Figure S5 Interaction of the 5′-A_0_ fragment and Rrp12 in wild type cells. Northern blot analysis showing copurification (second to bottom panels on the right) of the indicated pre-rRNA species and the 5′-A_0_ fragment with the indicated GFP-tagged proteins in normal cells. As control, a parallel Northern blot analysis was performed on total RNAs prepared from the same total cell lysate samples used for the GFP-Trap protein purifications (second to bottom panels on the left). Western blot experiments were performed to analyze the amounts of the GFP-tagged proteins present in the total cell lysates (top panel on the left) and in the purifications (top panel on the right). The strains used in this experiment were W303 (control), JDY851 (*nop7-GFP*) and YPM7-R (*GAL::HA-rrp12* containing a pRS416-GFP-rrp12 plasmid). These strains were maintained continuously in glucose-containing media.
(TIF)

Table S1 Yeast strains used in this study.
(PDF)

Table S2 Plasmids used in this study.
(PDF)

Table S3 Probes used in northern blot analysis.
(PDF)
