# Supplementary figures and images for: Rrp12 and the Exportin Crm1 Participate in Late Assembly Events in the Nucleolus during 40S Ribosomal Subunit Biogenesis
Source: PLoS Genet. 2014 Dec 4;10(12):e1004836. doi: 10.1371/journal.pgen.1004836 (PMC4256259; doi:10.1371/journal.pgen.1004836)

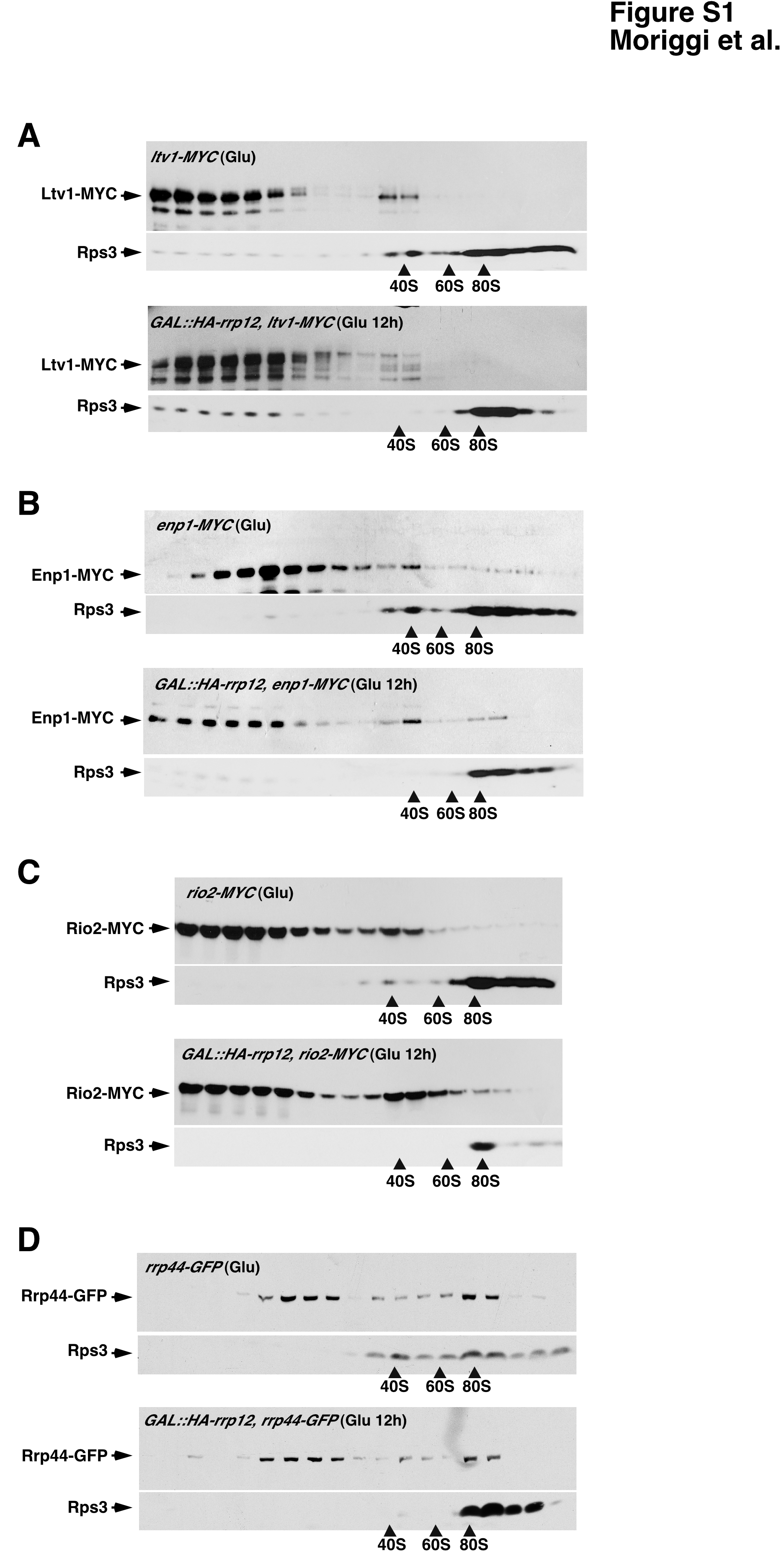

Supplement: Figure S1 — Recruitment of maturation factors to pre-40S particles in the absence of Rrp12. (A–C) Sucrose gradient analysis showing the sedimentation behavior of Ltv1-MYC (A), Enp1-MYC (B), Rio2-MYC (C) and Rrp44-GFP (D) in the presence (top two panels) and absence of Rrp12 (bottom two panels). Each set of gradient fractions was analyzed by Western blot with anti-MYC (A, B and C) of anti-GFP (D), and anti-Rps3. The positions of the gradient where 40S, 60S and 80S complexes sedimented are indicated by arrows. (TIF) [file pgen.1004836.s001.tif]

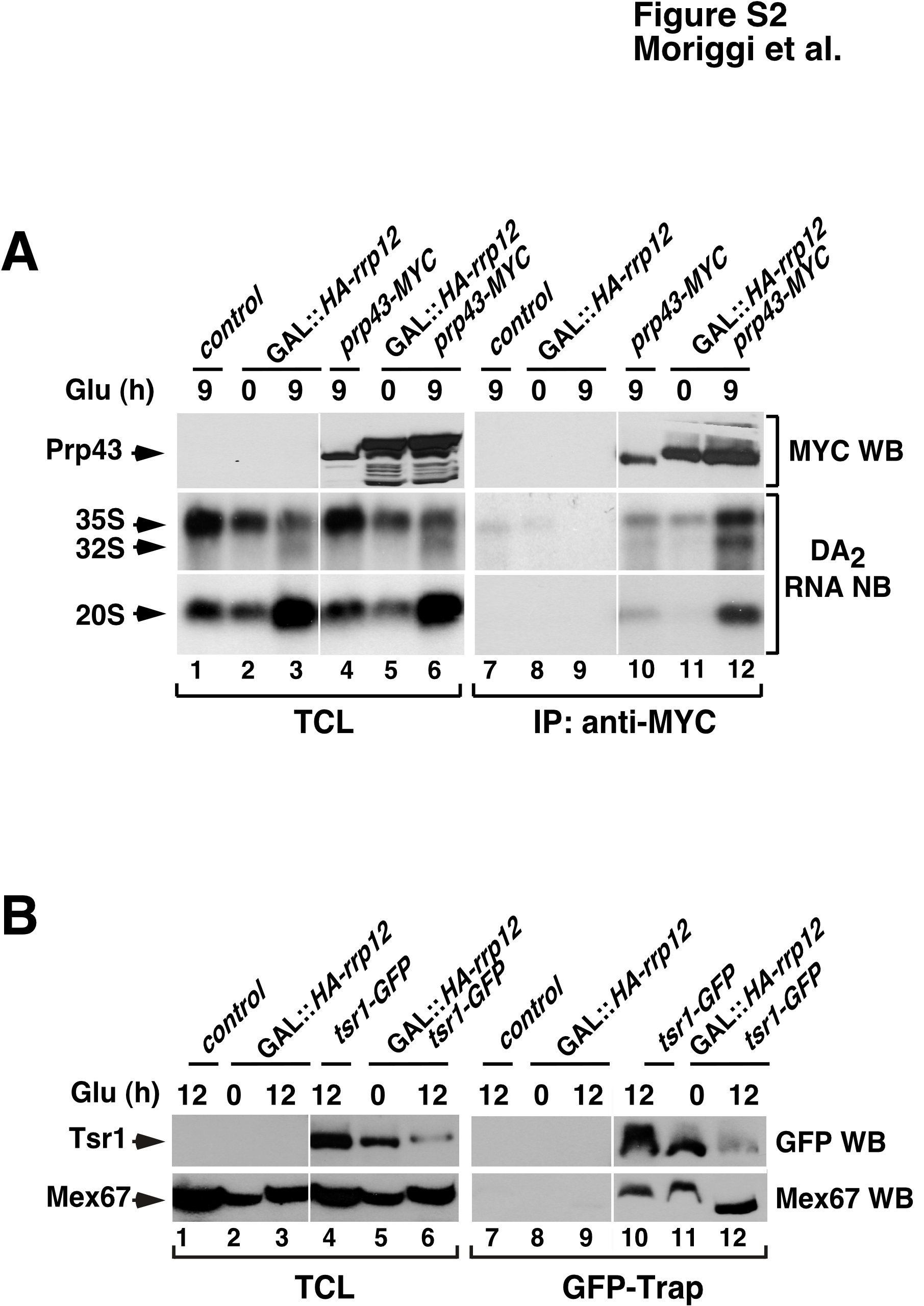

Supplement: Figure S2 — Rrp12 is not required for the association of Prp43 and Mex67 with pre-40S particles. (A) Northern blot analysis showing coimmunoprecipitation of the indicated pre-RNA species with Prp43-MYC in the presence and absence of Rrp12. Total RNAs (middle and bottom panels, lanes 1 to 6) and RNAs present in Prp43-MYC immunoprecipitates (middle and bottom panels, lanes 7 to 12) obtained from the indicated strains, grown under the indicated conditions, were analyzed with a probe that maps to the pre-rRNA D-A2 region. Western blot experiments were performed to analyze the amount of Prp43-MYC present in the total cell lysates (top panels, lanes 1 to 6) and immunoprecipitations (top panels, lanes 7 to 12). (B) Western blot analysis showing copurification of Mex67 with Tsr1-GFP in the presence and absence of Rrp12. Total cell lysates (lanes 1 to 6) and GFP-Trap purified complexes (lanes 7 to 12) obtained from the indicated yeast strains, grown under the indicated conditions, were analyzed with anti-MYC and anti-Mex67 antibodies. The thin white lines between lanes 3 and 4, and lanes 9 and 10, shown in A and B, indicate the presence of in-between lanes in the same blot that have been removed. (TIF) [file pgen.1004836.s002.tif]

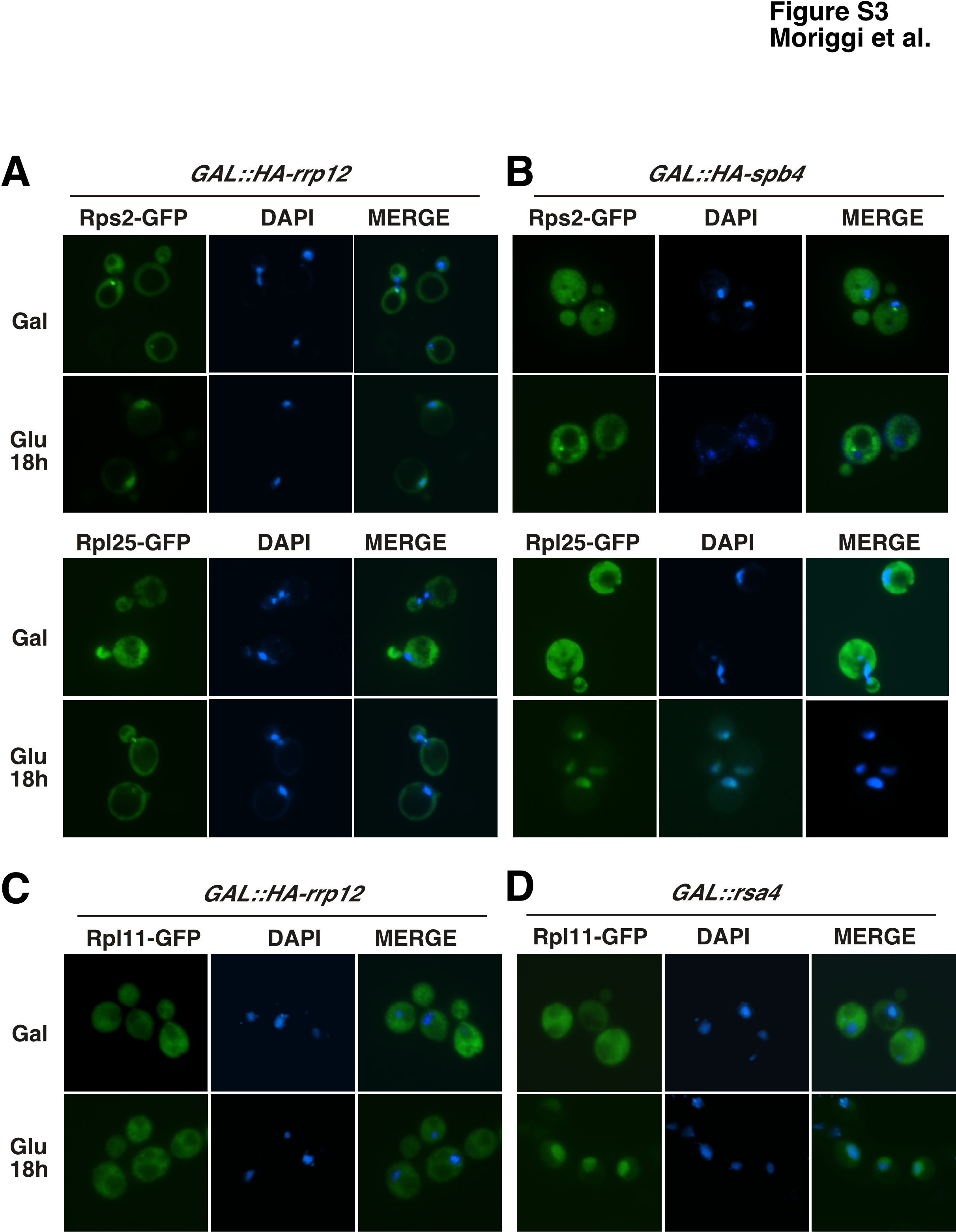

Supplement: Figure S3 — The loss of Rrp12 causes accumulation of pre-40S, but not pre-60S, complexes in the nucleus. Epifluorescence microscopy analysis of GAL::HA-rrp12 cells (A, C), control GAL::HA-spb4 cells (B), and control GAL::HA-rsa4 cells (D) expressing 40S (Rps2-GFP; top and second panels in A and B), 60S (Rpl25-GFP, third and bottom panels in A and B; and Rpl11-GFP, top and bottom panels in C and D) subunit reporters. These cells were grown in galactose-containing medium or shifted to glucose-containing medium for 18 h as indicated. The GFP signal, the DAPI-stained nuclei and the GFP-DAPI merge are shown in the left, middle and right panels, respectively. (TIF) [file pgen.1004836.s003.tif]

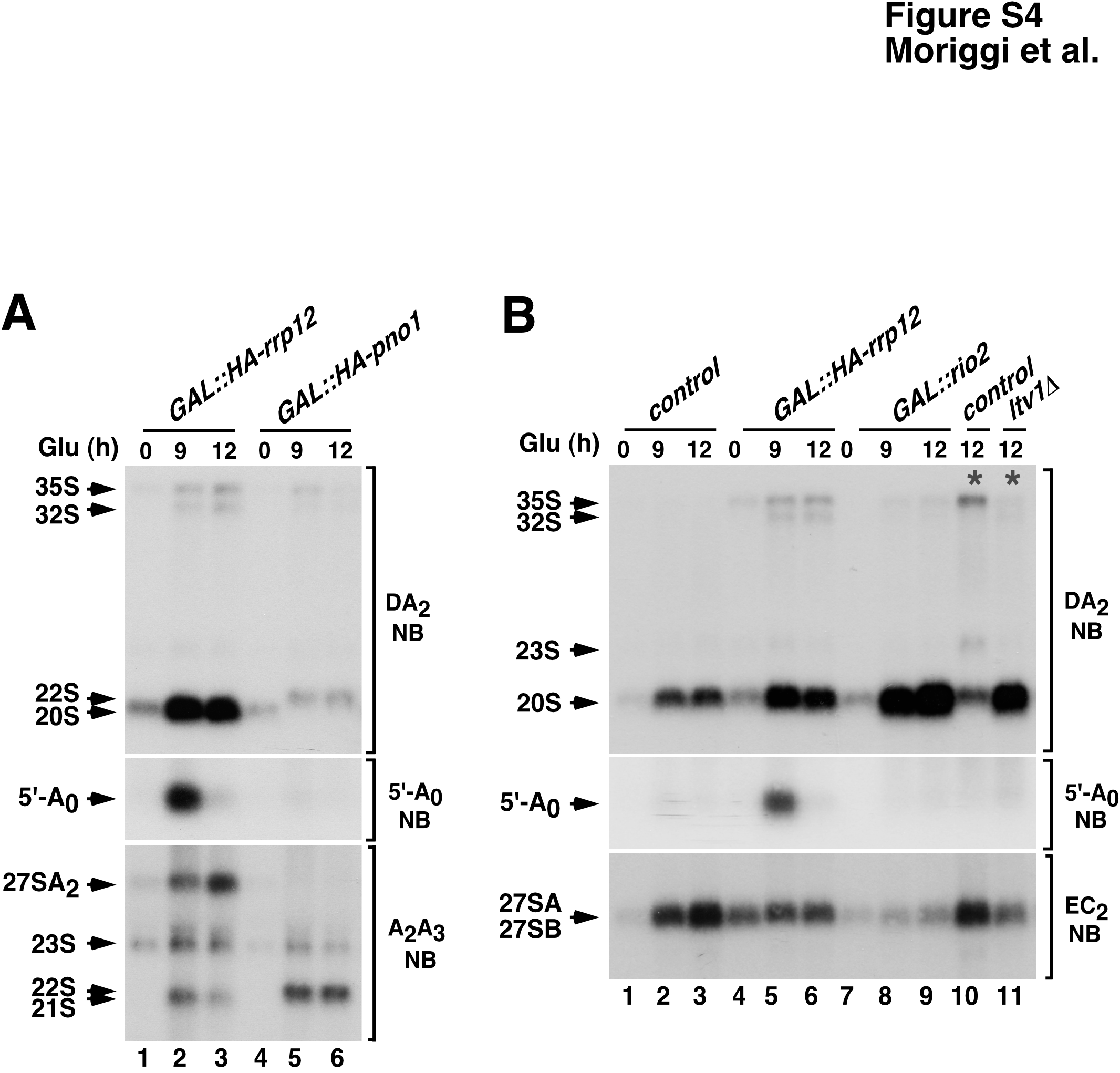

Supplement: Figure S4 — The loss of Pno1, Rio2 or Ltv1 does not cause accumulation of the 5′-A0 fragment. Northern blot analysis of total RNAs extracted from GAL::HA-rrp12 and GAL::HA-pno1 cells (A), and from GAL::HA-rrp12, GAL::rio2 and ltv1Δ cells (B). Cells were grown at 30°C (except those corresponding to the lanes marked with an asterisk in B) in galactose-containing media or shifted to glucose-containing media for the indicated times. The samples marked with an asterisk (lanes 10 and 11 in B) were prepared from cultures grown at 25°C, the temperature at which the defects of the LTV1 deletion are most patent. The specific region of the 35S pre-rRNA recognized by each Northern blot probe is indicated on the right. (TIF) [file pgen.1004836.s004.tif]

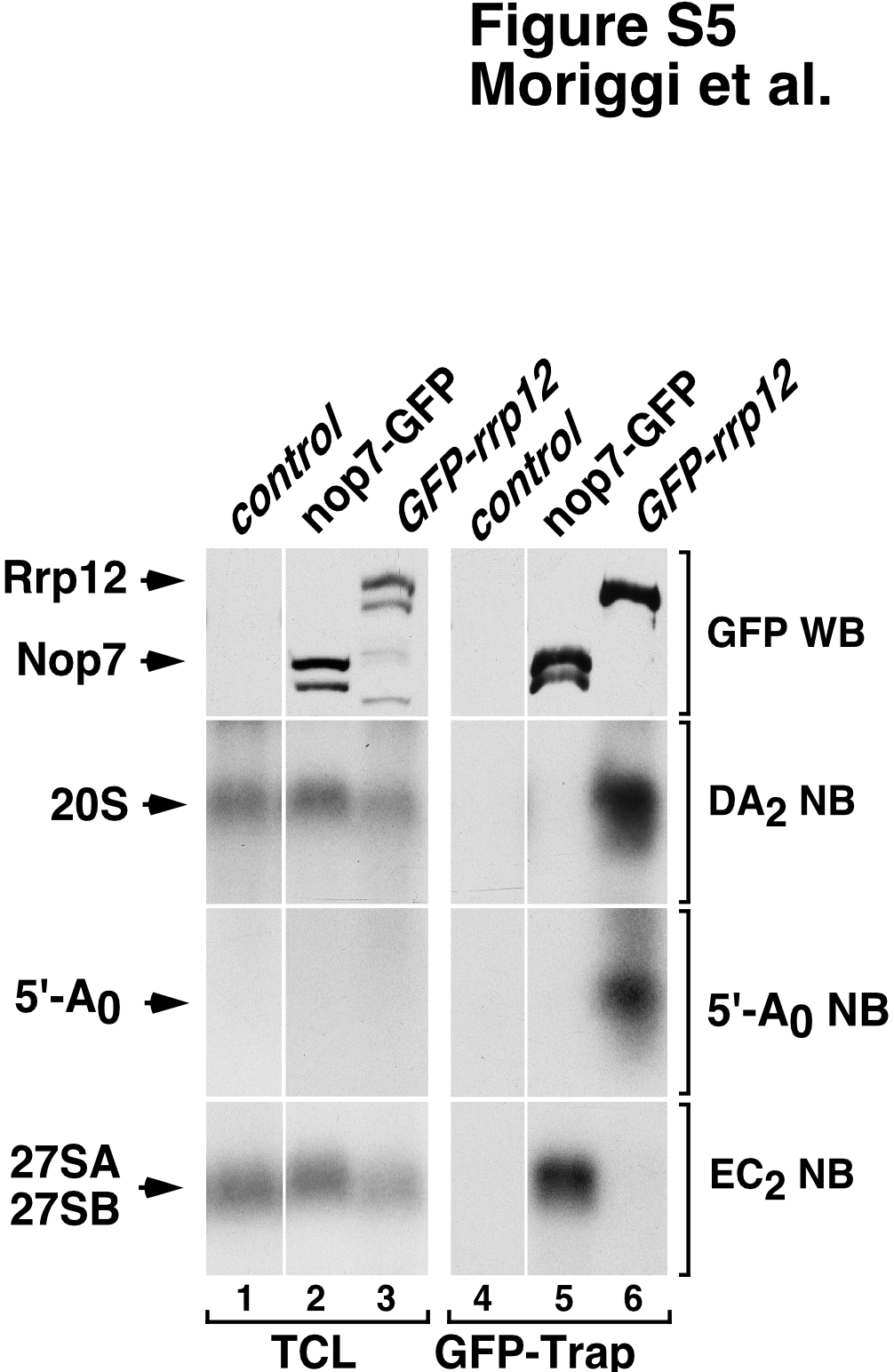

Supplement: Figure S5 — Interaction of the 5′-A0 fragment and Rrp12 in wild type cells. Northern blot analysis showing copurification (second to bottom panels on the right) of the indicated pre-rRNA species and the 5′-A0 fragment with the indicated GFP-tagged proteins in normal cells. As control, a parallel Northern blot analysis was performed on total RNAs prepared from the same total cell lysate samples used for the GFP-Trap protein purifications (second to bottom panels on the left). Western blot experiments were performed to analyze the amounts of the GFP-tagged proteins present in the total cell lysates (top panel on the left) and in the purifications (top panel on the right). The strains used in this experiment were W303 (control), JDY851 (nop7-GFP) and YPM7-R (GAL::HA-rrp12 containing a pRS416-GFP-rrp12 plasmid). These strains were maintained continuously in glucose-containing media. (TIF) [file pgen.1004836.s005.tif]
