# Supplementary material for: Rrp12 and the Exportin Crm1 Participate in Late Assembly Events in the Nucleolus during 40S Ribosomal Subunit Biogenesis
Source: PLoS Genet. 2014 Dec 4;10(12):e1004836. doi: 10.1371/journal.pgen.1004836 (PMC4256259; doi:10.1371/journal.pgen.1004836)
Supplement: Table S1 — Yeast strains used in this study. (PDF) [file pgen.1004836.s006.pdf]

TABLE S1. **Yeast strains used in this study**

Name	Genotype	Source
W303a	*MATa, ade2, his3, leu2, trp1, ura3*	Euroscarf
JDY144	*ade2, his3, leu2, ura3, spb4Δ::TRP [pGAL::HA-spb4 LEU2]*	[1]
JDY207	*MATa, his3, leu2, trp1, ura3, mtr4Δ::HIS3MX [pYCplac33-MTR4 URA3]*	[2]
JDY850	*MATa, ade2, his3, leu2, trp1, ura3, ssf1-GFP(S65T)::natNT2*	[3]
JDY851	*MATa, ade2, his3, leu2, trp1, ura3, nop7-GFP(S65T)::natNT2*	[3]
JDY852	*MATa, ade2, his3, leu2, trp1, ura3, rix1-GFP(S65T)::natNT2*	[3]
JDY853	*MATa, ade2, his3, leu2, trp1, ura3, kre35-GFP(S65T)::natNT2*	[3]
JDY855	*MATa, ade2, his3, leu2, trp1, ura3, arx1-GFP(S65T)::natNT2*	[3]
MNY7	*MATa, ade2, his3, leu2, trp1, ura3, crm1Δ::KANMX6 [pDC-CRM1 LEU2]*	[4]
MNY8	*MATa, ade2, his3, leu2, trp1, ura3, crm1Δ::KANMX6 [pDC-crm1 (T539C) LEU2]*	[4]
WDG72	*MATa, ade2, leu2, ura, rsa4Δ::URA3 [pGAL::HA-rsa4 TRP1]*	[5]
YBN11	*MATa, ade2, leu2, trp1, ura3, KANMX-GAL::HA-rrp12, rrp44-GFP(S65T)::HIS3MX*	This study
YBN13	*MATa, ade2, leu2, trp1, ura3, rrp44- GFP(S65T)::HIS3MX*	This study
YGM1	*MATa, ade2, his3, leu2, trp1, ura3, tsr1-GFP(S65T)::TRP1*	This study
YGM6	*MATa, ade2, his3, leu2, trp1, ura3, tsr1-MYC::HIS3MX*	This study
YGM92	*MATa, ade2, his3, leu2, trp1, ura3, KANMX-GAL::HA-rrp12, nob1-GFP(S65T)::HIS3MX*	This study
YGM94	*MATa, ade2, his3, leu2, trp1, ura3, nob1-GFP(S65T)::HIS3MX*	This study
YGM96	*MATa, ade2, his3, leu2, trp1, ura3, enp1-GFP(S65T)::HIS3MX*	This study
YGM93	*MATa, ade2, his3, leu2, trp1, ura3, KANMX-GAL::HA-rrp12*	This study
YGM98	*MATa, ade2, his3, leu2, trp1, ura3, KANMX-GAL::HA-rrp12, pwp2-GFP(S65T):: HIS3MX*	This study
YGM99	*ade2, his3, leu2, trp1, ura3, KANMX-GAL::HA-rrp12, nop7-GFP(S65T)::natNT2*	This study
YGM102	*MATa, ade2, his3, leu2, trp1, ura3, KANMX-GAL::HA-rrp12, enp1-GFP(S65T)::HIS3MX*	This study

YGM104	*MATa, ade2, his3, leu2, trp1, ura3, KANMX-GAL::HA-rrp12, tsr1-GFP(S65T)::TRP1*	This study
YGM119	*MATa, ade2, his3, leu2, ura3, ltv1Δ::TRP1*	This study
YGM143	*MATa, ade2, his3, leu2, trp1, ura3, prp43-MYC::HIS3MX*	This study
YGM145	*MATa, ade2, his3, leu2, trp1, ura3, ltv1-MYC::HIS3MX*	This study
YGM147	*MATa, ade2, his3, leu2, trp1, ura3, KANMX-GAL::HA-rrp12, prp43-MYC::HIS3MX*	This study
YGM148	*MATa, ade2, his3, leu2, trp1, ura3, KANMX-GAL::HA-rrp12, ltv1-MYC::HIS3MX*	This study
YGM149	*MATa, ade2, his3, leu2, trp1, ura3, enp1-MYC::HIS3MX*	This study
YGM151	*MATa, ade2, his3, leu2, trp1, ura3, rio2-MYC::HIS3MX*	This study
YGM152	*MATa, ade2, his3, leu2, trp1, ura3, dim1-MYC::HIS3MX*	This study
YGM154	*MATa, ade2, his3, leu2, trp1, ura3, KANMX-GAL::HA-rrp12, rio2-MYC::HIS3MX*	This study
YGM155	*MATa, ade2, his3, leu2, trp1, ura3, KANMX-GAL::HA-rrp12, enp1-MYC::HIS3MX*	This study
YGM156	*MATa, ade2, his3, leu2, trp1, ura3, KANMX-GAL::HA-rrp12, dim1-MYC::HIS3MX*	This study
YGM168	*MATa, ade2, his3, leu2, trp1, ura3, KANMX-GAL::HA-pno1*	This study
YGM174	*MATa, leu2, his3, trp1, ura3, mtr4Δ::HIS3MX [pGAL::HA-mtr4 LEU2]*	This study
YGM193	*MATa, ade2, his3, leu2, trp1, ura3, pwp2-GFP(S65T)::HIS3MX, crm1Δ::KANMX6 [pDC-CRM1 LEU2]*	This study
YLG1	*MATa, ade2, his3, leu2, trp1, ura3, rio2-GFP(S65T)::HIS3MX*	This study
YLG2	*MATa, ade2, his3, leu2, trp1, ura3, ltv1-GFP(S65T)::HIS3MX*	This study
YLG5	*MATa, ade2, his3, leu2, trp1, ura3, KANMX-GAL::HA-rrp12, rio2-GFP(S65T)::HIS3MX*	This study
YLG7	*MATa, ade2, his3, leu2, trp1, ura3, KANMX-GAL::HA-rrp12, dim1-GFP(S65T)::HIS3MX*	This study
YLG9	*MATa, ade2, his3, leu2, trp1, ura3, dim1-GFP(S65T)::HIS3MX*	This study
YLG11	*MATa, ade2, his3, leu2, trp1, ura3, pno1-GFP(S65T)::HIS3MX*	This study
YLG13	*MATa, ade2, his3, leu2, trp1, ura3, KANMX-GAL::HA-rrp12, pno1-GFP(S65T)::HIS3MX*	This study
YMD6	*MATa, ade2, his3, leu2, trp1, ura3, pwp2-GFP(S65T)::KANMX*	This study
YMD44	*MATa, ade2, his3, leu2, trp1, ura3, nop7-MYC::HIS3MX*	[5]

YMD57	*MATa, ade2, his3, leu2, trp1, ura3, pwp2-MYC::HIS3MX*	[5]
YMD230	*MATa, ade2, his3, leu2, trp1, ura3, rrp12-MYC::KANMX*	[6]
YMD392	*MATa, ade2, his3, leu2, trp1, ura3, hrr25-GFP(S65T)::HIS3MX*	This study
YMD393	*MATa, ade2, his3, leu2, trp1, ura3, KANMX-GAL::HA-rrp12, ltv1-GFP(S65T)::HIS3MX*	This study
YO470	*MATa, ade2, his3, leu2, trp1, ura3, rio2Δ:: KANMX6 [2μ pGAL::rio2-PROTA URA]*	[7]
YPM7	*MATa, ade2, his3, leu2, trp1, ura3, KANMX-GAL::HA-rrp12*	This study
YPM7-R	*MATa, ade2, his3, leu2, trp1, ura3, KANMX-GAL::HA-rrp12 [pGM57]*	This study

1. de la Cruz J, Kressler D, Rojo M, Tollervey D, Linder P (1998) Spb4p, an essential putative RNA helicase, is required for a late step in the assembly of 60S ribosomal subunits in Saccharomyces cerevisiae. RNA 4: 1268-1281.

2. de la Cruz J, Kressler D, Tollervey D, Linder P (1998) Dob1p (Mtr4p) is a putative ATP-dependent RNA helicase required for the 3' end formation of 5.8S rRNA in Saccharomyces cerevisiae. EMBO J 17: 1128-1140.

3. Garcia-Gomez JJ, Lebaron S, Froment C, Monsarrat B, Henry Y, et al. (2011) Dynamics of the putative RNA helicase Spb4 during ribosome assembly in Saccharomyces cerevisiae. Mol Cell Biol 31: 4156-4164.

4. Neville M, Rosbash M (1999) The NES-Crm1p export pathway is not a major mRNA export route in Saccharomyces cerevisiae. EMBO J 18: 3746-3756.

5. Dosil M, Bustelo XR (2004) Functional characterization of Pwp2, a WD family protein essential for the assembly of the 90 S pre-ribosomal particle. J Biol Chem 279: 37385-37397.

6. Dosil M (2011) Ribosome synthesis-unrelated functions of the preribosomal factor Rrp12 in cell cycle progression and the DNA damage response. Mol Cell Biol 31: 2422-2438.

7. Vanrobays E, Gelugne JP, Gleizes PE, Caizergues-Ferrer M (2003) Late cytoplasmic maturation of the small ribosomal subunit requires RIO proteins in Saccharomyces cerevisiae. Mol Cell Biol 23: 2083-2095.
