# Supplementary material for: Rrp12 and the Exportin Crm1 Participate in Late Assembly Events in the Nucleolus during 40S Ribosomal Subunit Biogenesis
Source: PLoS Genet. 2014 Dec 4;10(12):e1004836. doi: 10.1371/journal.pgen.1004836 (PMC4256259; doi:10.1371/journal.pgen.1004836)
Supplement: Table S2 — Plasmids used in this study. (PDF) [file pgen.1004836.s007.pdf]

TABLE S2. **Plasmids used in this study**

Name	Relevant information	Source
pBN18	CEN, HIS3, RRP12	This study
pBN19	CEN, HIS3, rrp12 (198-1228 aa)	This study
pDC-CRM1	CEN, LEU2, CRM1	[1]
pDC-crm1(T539C)	CEN, LEU2, crm1 (T539C)	[1]
pGM57	CEN, URA3, GFP-rrp12	This study
pGM58	CEN, HIS3, GFP-rrp12	This study
pLG1	CEN, LEU2, RRP12	This study
pLG2	CEN, LEU2, rrp12 (198-1228 aa)	This study
pRS316-RPS2-GFP	CEN, URA3, rps2-GFP	[2]
pRS316-RPL25-GFP	CEN, URA3, rpl25-GFP	[3]
pRPL11-GFP	CEN, LEU2, rpl11-GFP	Jesús de la Cruz

1. Neville M, Rosbash M (1999) The NES-Crm1p export pathway is not a major mRNA export route in Saccharomyces cerevisiae. EMBO J 18: 3746-3756.

2. Milkereit P, Strauss D, Bassler J, Gadal O, Kuhn H, et al. (2003) A Noc complex specifically involved in the formation and nuclear export of ribosomal 40 S subunits. J Biol Chem 278: 4072-4081.

3. Gadal O, Strauss D, Kessl J, Trumpower B, Tollervey D, et al. (2001) Nuclear export of 60s ribosomal subunits depends on Xpo1p and requires a nuclear export sequence-containing factor, Nmd3p, that associates with the large subunit protein Rpl10p. Mol Cell Biol 21: 3405-3415.
