# Supplementary material for: Rrp12 and the Exportin Crm1 Participate in Late Assembly Events in the Nucleolus during 40S Ribosomal Subunit Biogenesis
Source: PLoS Genet. 2014 Dec 4;10(12):e1004836. doi: 10.1371/journal.pgen.1004836 (PMC4256259; doi:10.1371/journal.pgen.1004836)
Supplement: Table S3 — Probes used in northern blot analysis. (PDF) [file pgen.1004836.s008.pdf]

TABLE S3. **Probes used in Northern blot analysis**

**Probe (region recognized)**	**Sequence (5'-3')**
Probe 01 (5’-A_0)	TCAGGTCTCTGCTGC
Probe 02 (18S)	AGCCATTCGCAGTTTCACTG
Probe 03 (D-A_2)	TTAAGCGCAGGCCCGGCT
Probe 04 (A_2-A_3)	TGTTACCTCTGGGCC
Probe 05 (5.8S)	GCGTTCTTCATCGATGC
Probe 06 (5’E-C_2)	TGAGAAGGAAATGACGCT
Probe 07 (E-C_2)	GGCCAGCAATTTCAAGTTA
Probe 08 (25S)	TACTAAGGCAATCCCGGTTGG
U3	GGATTGCGGACCAAGCTAA
scR1	ATCCCGGCCGCCTCCATCAC
